# Understanding glycaemic control and current approaches for screening antidiabetic natural products from evidence-based medicinal plants

**DOI:** 10.1186/s13007-019-0487-8

**Published:** 2019-09-07

**Authors:** Chintha Lankatillake, Tien Huynh, Daniel A. Dias

**Affiliations:** 1 School of Health and Biomedical Sciences, Discipline of Laboratory Medicine, RMIT University, Bundoora, 3083 Australia; 2 School of Science, RMIT University, Bundoora, VIC 3083 Australia

**Keywords:** Antidiabetic, Blood glucose, Chemical profiling, Diabetes mellitus, Experimental models, Glucose uptake, Insulin resistance, Medicinal plants, Metabolomics, Natural products

## Abstract

Type 2 Diabetes Mellitus has reached epidemic proportions as a result of over-nutrition and increasingly sedentary lifestyles. Current therapies, although effective, are not without limitations. These limitations, the alarming increase in the prevalence of diabetes, and the soaring cost of managing diabetes and its complications underscores an urgent need for safer, more efficient and affordable alternative treatments. Over 1200 plant species are reported in ethnomedicine for treating diabetes and these represents an important and promising source for the identification of novel antidiabetic compounds. Evaluating medicinal plants for desirable bioactivity goes hand-in-hand with methods in analytical biochemistry for separating and identifying lead compounds. This review aims to provide a comprehensive summary of current methods used in antidiabetic plant research to form a useful resource for researchers beginning in the field. The review summarises the current understanding of blood glucose regulation and the general mechanisms of action of current antidiabetic medications, and combines knowledge on common experimental approaches for screening plant extracts for antidiabetic activity and currently available analytical methods and technologies for the separation and identification of bioactive natural products. Common in vivo animal models, in vitro models, in silico methods and biochemical assays used for testing the antidiabetic effects of plants are discussed with a particular emphasis on in vitro methods such as cell-based bioassays for screening insulin secretagogues and insulinomimetics. Enzyme inhibition assays and molecular docking are also highlighted. The role of metabolomics, metabolite profiling, and dereplication of data for the high-throughput discovery of novel antidiabetic agents is reviewed. Finally, this review also summarises sample preparation techniques such as liquid–liquid extraction, solid phase extraction, and supercritical fluid extraction, and the critical function of nuclear magnetic resonance and high resolution liquid chromatography–mass spectrometry for the dereplication, putative identification and structure elucidation of natural compounds from evidence-based medicinal plants.

## Background

Ancient physicians believed diabetes to be a rare condition of the kidneys and bladder which led to copious production of urine (polyuria) and excessive thirst (polydipsia) ultimately resulting in the death of the patient [1, 2]. Presently, our understanding of diabetes has much improved aided by the tenacious work of physicians and scientists. Diabetes is now recognised as a chronic metabolic disease caused by relative insulin deficiency, reduced insulin sensitivity of body tissues, or a combination thereof. The disease is complex in aetiology, involves multiple organ systems, and is characterised by impaired blood glucose control [3].

**Fig. 1:**
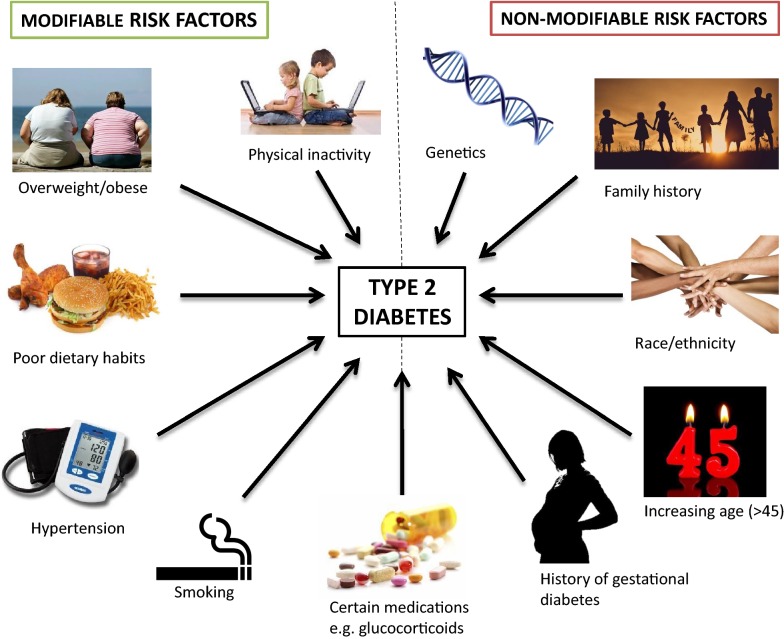
Modifiable and non-modifiable risk factors of Type 2 Diabetes Mellitus

There are two main clinical classes of diabetes. Type 1 Diabetes Mellitus (T1DM) accounts for 5–10% of all cases of diabetes and is caused by the autoimmune destruction of pancreatic *β*-cells which results in insulin deficiency. On the other hand, Type 2 Diabetes Mellitus (T2DM) results from the interaction between genetic predisposition and modifiable and non-modifiable environmental risk factors such as obesity, poor diet and lack of exercise (Fig. 1). Insulin resistance and the progressive deterioration of pancreatic *β*-cells are central to the aetiology of T2DM [4]. T2DM is the most common form of the disease (90–95% of all cases) and is therefore the major focus of diabetes research.

Hyperglycaemia (fasting blood glucose level ≥ 7.0 mmol/L, or random blood glucose level ≥ 11.1 mmol/L) is the physiological anomaly characteristic of diabetes. Therefore, measuring blood glucose level (BGL) is used for screening, diagnosing and monitoring diabetes [5]. There are four types of tests available: (1) fasting blood glucose, (2) oral glucose tolerance test, (3) random glucose test, and (4) HbA1c, or glycated haemoglobin test [5, 6].

**Fig. 2:**
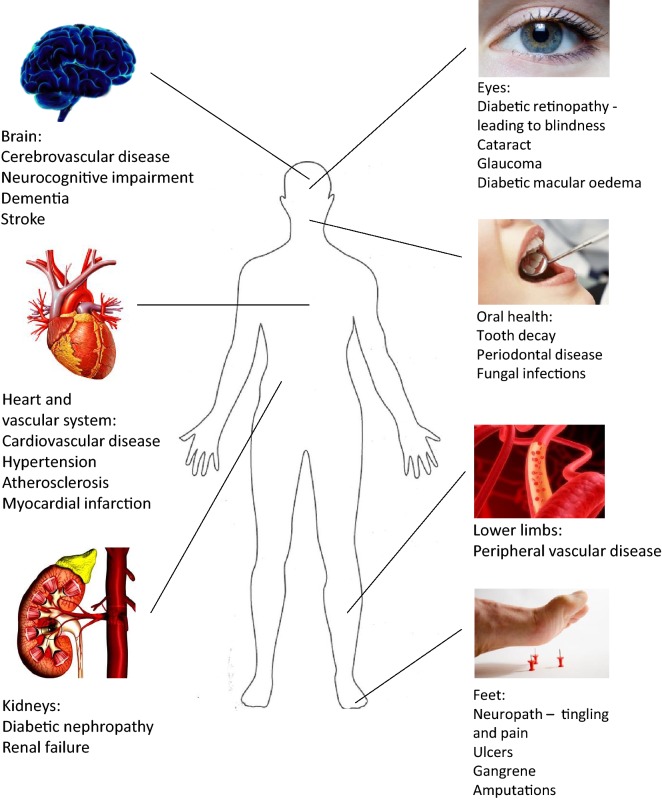
Long-term complications of diabetes

Sustained high blood glucose levels (such as in the case of diabetes) is damaging to many organ systems. Therefore, T2DM is associated with severe long-term complications (Fig. 2) which contributes to high levels of morbidity and mortality. For example, vascular and nerve damage caused by chronic hyperglycaemia hastens the progression of cardiovascular disease (CVD), and can lead to vision loss and renal failure [7]. Diabetes-induced retinopathy is the primary cause of preventable blindness, while diabetic nephropathy is a major contributor to kidney disease [8]. Therefore, the appropriate management of hyperglycaemia is a key goal of diabetes treatment and aims to minimise the development of chronic complications.

Direct expenditure required for the long-term management of diabetes and its complications is a significant burden on national economies. Indirect costs related to premature mortality, and the loss of productivity as demonstrated by labour-market studies, also contributes to the economic impact of diabetes [8, 9]. It is estimated that 12% of global healthcare expenditure is spent on diabetes [10]. The global prevalence of diabetes has reached epidemic proportions, and with worldwide incidence projected to increase from 424 million in 2017 to 629 million by 2045 [11], the global economic burden of the disease can be expected to skyrocket. Therefore, there is a pressing need to invest in research of novel therapies for the treatment and management of diabetes.

The scientific investigation of plants recognised for their medicinal value is an alternate and effective strategy for the discovery of novel therapeutic agents [12, 13]. For example, 1,1-dimethylbiguanide, the active ingredient in the well-known antidiabetic drug metformin, is derived from guanidine; an antihyperglycaemic compound discovered from the French lilac plant, *Galega officinalis* [14]. Some commonly prescribed conventional medicines were also discovered from plants. Popular examples include the analgesic morphine from *Papaver somniferum* (poppy), the anti-inflammatory drug aspirin from *Salix alba* (white willow), and the anti-malarial drug, quinine from *Cinchona succirubra* [12]. Such examples demonstrate that scientific investigation into evidence-based medicinal plants for discovering bioactive natural products (NPs) with therapeutic applications is a promising avenue for establishing novel therapies for disease.

Evaluating medicinal plants for antidiabetic properties goes hand-in-hand with methods in analytical biochemistry for separating and identifying bioactive compounds. This review aims (1) to summarise the current understanding of blood glucose regulation and the general mechanisms of action of current antidiabetic medications and (2) to combine knowledge on common experimental approaches including in vivo animal models, in vitro cell models, and biochemical assays for screening and testing plants for antidiabetic activity, and currently available analytical methods and technologies for the separation and identification of bioactive NPs.

## Blood glucose homeostasis

The search for antidiabetic agents requires an understanding of how blood glucose levels are regulated in the body, and the general mechanisms of action of current antidiabetic therapies.

Blood glucose homeostasis in the body is maintained by two antagonistic hormones: insulin and glucagon. Insulin and glucagon are produced by endocrine cells located in the islets of Langerhans of the pancreas. Insulin is secreted by pancreatic *β*-cell in the fed-state and is responsible for lowering blood glucose levels following a meal or when blood glucose levels are high [15]. On the contrary, glucagon is secreted by pancreatic *α*-cells during the fasted-state and acts to raise blood glucose levels. Together, these hormones maintain blood glucose levels within the normal range (normoglycaemia) [16]. In T2DM, insulin is given higher importance over glucagon because defective insulin production/function and/or the body’s response to insulin is at the heart of the pathology of T2DM. In addition, the physiological consequences of defective insulin secretion or function are far more detrimental than that of glucagon dysfunction.

### Insulin and the insulin receptor

**Fig. 3:**
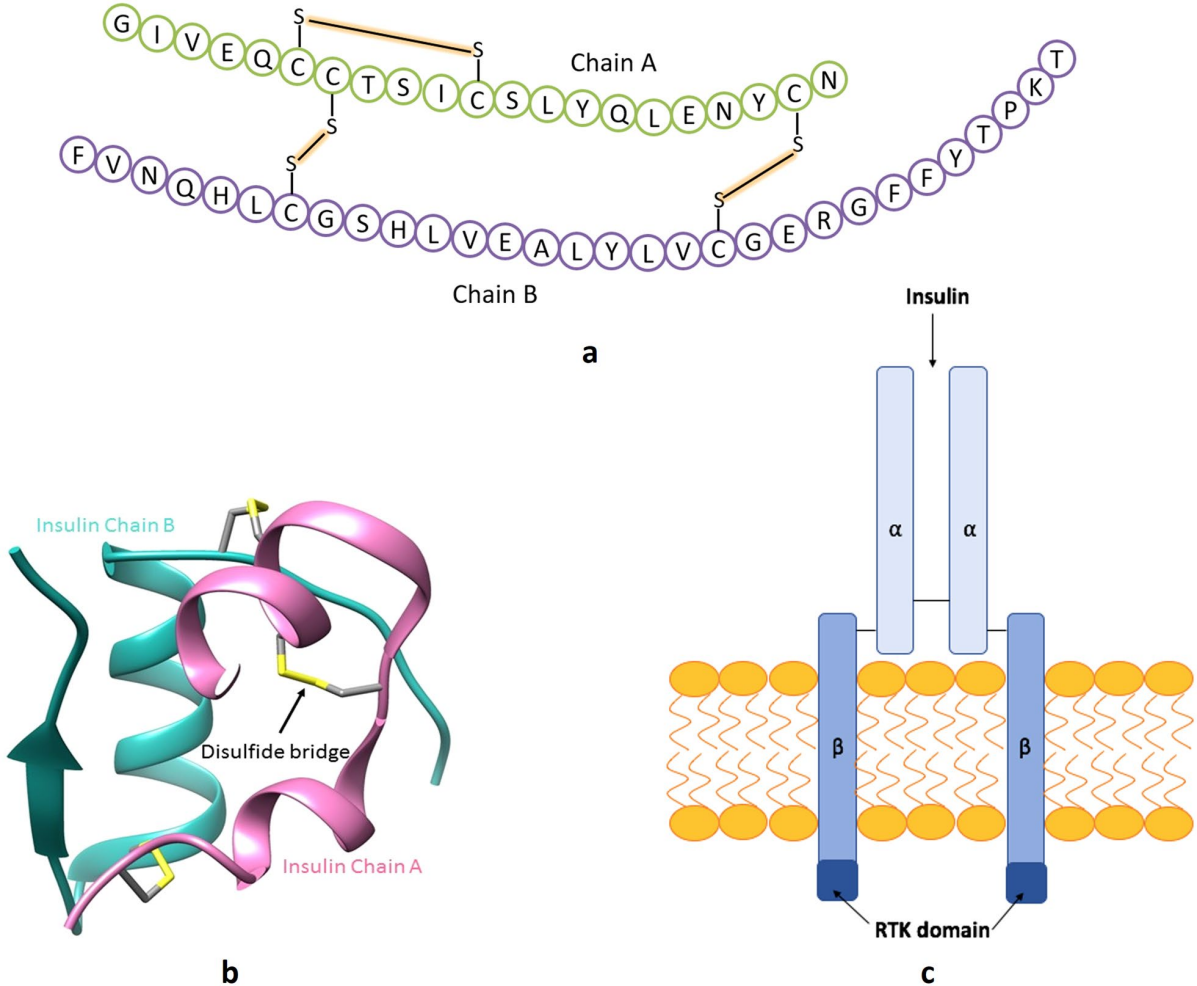
**a** Human insulin: amino acid sequence,** b** 3-dimensional structure (image from [299]),** c** simplified structure of the insulin receptor

Insulin is an anabolic peptide hormone, the structure of which consists of a two-chain heterodimer composed of a 21-residue A-chain and a 30-residue B-chain linked together with two disulphide bonds formed between cysteine residues A7-B7 and A20-B19 (Fig. 3) [17].

Insulin produces its cellular effects by binding to a specific cell surface receptor and activating intracellular signal transduction cascades. The insulin receptor (INS-R) belongs to the receptor tyrosine kinase (RTK) superfamily of transmembrane proteins and is comprised of two extracellular *α*-subunits and two transmembrane *β*-subunits linked via disulphide bonds to form a *α*2*β*2 heterodimer (Fig. 3c) [15, 18, 19].

When insulin binds to the ligand-binding domain of the INS-R, the RTK domain is activated via a conformational change in the INS-R. This leads to autophosphorylation of the *β*-subunit on three tyrosine residues which act as a binding site for other messenger proteins containing src-homology 2 (SH2) domains or phosphotyrosine-binding (PTB) domains [20]. Phosphorylation of these proteins initiates signalling pathways resulting in various effects such as the expression of the GLUT4 (glucose transporter type 4) glucose transporter protein or activation of target enzymes or transcriptional factors. These effects are responsible for the metabolic effects of insulin [21].

### The role of insulin in blood glucose homeostasis

**Fig. 4:**
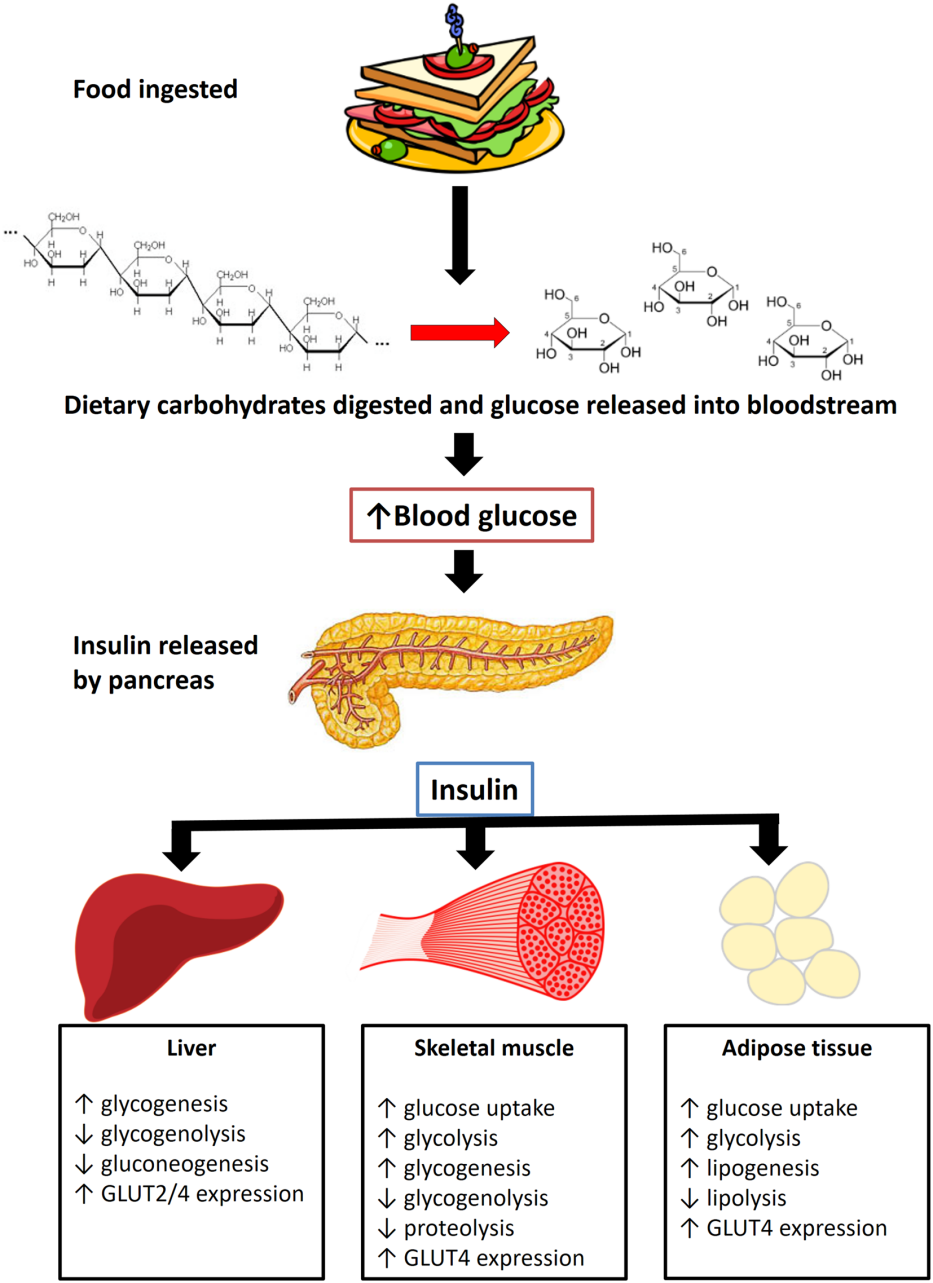
Effects of insulin on the major insulin-sensitive organs and tissues

The postprandial rise in blood glucose following the ingestion of a meal stimulates the release of insulin from pancreatic *β*-cells. Insulin instigates multiple actions in multiple tissues and organs in the body (Fig. 4). The three major sites of insulin action are the liver, muscle tissue and adipose tissue [22]. Insulin also acts on the pancreas to inhibit the secretion of glucagon [23]. Together, the action of insulin on the liver, muscle tissue and adipose tissue result in the removal of glucose from the blood and the restoration of normal blood glucose levels.

#### Liver

Insulin promotes the synthesis of glycogen (storage form of carbohydrate) in the liver by activating glycogen synthase. As glucose is polymerised to form glycogen (glycogenesis), blood glucose levels decrease. Furthermore, insulin inhibits hepatic glucose production via gluconeogenesis by inhibiting gene expression of phosphoenolpyruvate carboxykinase (PEPCK) and glucose-6-phosphatase (G6Pase); the enzymes catalysing the rate-limiting step, and the final step of gluconeogenesis respectively [24, 25]. Insulin also suppresses glycogenolysis by glycogen phosphorylase to produce glucose [24]. Unlike muscle cells and adipocytes which express GLUT-4 transporters, hepatocytes mediate glucose uptake via GLUT-2 transporters. GLUT-2 transporters are not insulin-sensitive. Therefore, glucose uptake in liver cells occur independent of insulin and as a result, insulin has no direct influence on glucose uptake by hepatocytes [26].

#### Muscle

In muscle tissue, insulin promotes the translocation of GLUT4 transporters to the cell surface thereby enhancing insulin-stimulated glucose uptake by the cells. In addition, insulin increases glycolysis and glycogenesis and inhibits glycogenolysis and proteolysis. The increase in glucose uptake, glycolysis, and glycogenesis, and the decrease in glycogenolysis are responsible for lowering blood glucose levels [22, 27]. Glucose disposal by muscles is a major mechanism of controlling the postprandial rise in blood glucose [20].

#### Adipose tissue

Adipocytes also express the insulin-sensitive GLUT4 transporter. Therefore, insulin increases fatty tissue glucose uptake by enhancing the expression of GLUT4, which in turn increases glycolysis. Insulin also increases lipogenesis and inhibits lipolysis, which is beneficial for maintaining a healthy balance of serum lipids [22, 28, 29].

### Insulin-stimulated glucose uptake

**Fig. 5:**
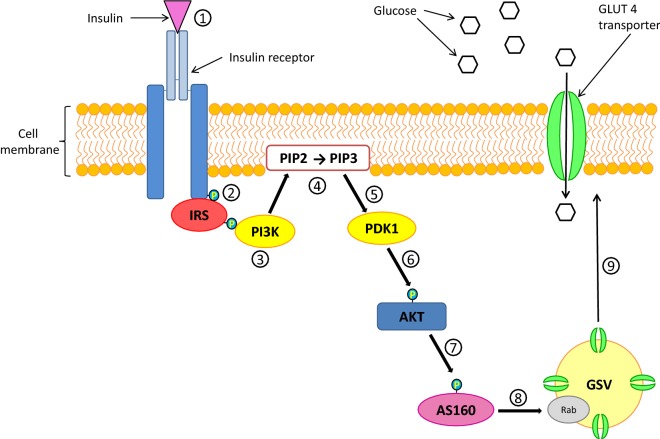
Insulin-stimulated GLUT4 trafficking via the PI3K/PDK1/Akt pathway

An important outcome of insulin receptor signalling is the translocation of the GLUT4 transporter to the cellular membrane, which facilitates the uptake of glucose by insulin-sensitive cells such as in adipocytes, cardiac muscles and skeletal muscles [20, 30]. Inside cells, GLUT4 transporters are sequestered within storage vesicles known as GLUT4 storage vesicles (GSV). In the presence of insulin, GSVs migrate to the cell surface and GLUT4 transporter proteins are expressed on the cell membrane via exocytosis [31]. This is accomplished via the PI3K/PDK1/Akt (phosphatidylinositol3-kinase/phosphoinositide-dependent protein kinase-1/protein kinase B) pathway [20]. The PI3K/PDK1/Akt pathway is briefly described below and illustrates the critical steps in the pathway (Fig. 5).

Firstly, insulin binds to its receptor causing the activation of the RTK domain and the activated RTK domain auto-phosphorylates key tyrosine residues on its external surface **(1)** [32]. The insulin RTK phosphorylates specific tyrosine residues on the insulin receptor substrate (IRS) proteins **(2)**. These phosphorylated tyrosine residues serve as docking sites for proteins containing a SH2 or PTB (protein tyrosine-binding) domains [22]. The SH2 domain on the p85 regulatory subunit of PI3K binds to tyrosine-phosphorylated IRS activating the p110 catalytic domain on PI3K **(3)**. The activated PI3K is recruited to the cell membrane and p110 converts phosphatidylinositol-4,5-*bi*phosphate (PIP2) to phosphatidylinositol-3,4,5-*tri*phosphate (PIP3) **(4)** [20]. PIP3 up-regulates 3-phosphatidylinositol-dependent kinase-1 (PDK1) activity **(5)**, where PDK1 phosphorylates and activates protein kinase B (Akt) **(6)** [24].

Akt phosphorylates the Rab GTPase-activating protein AS160. Phosphorylation inactivates AS160 **(7)**. Rab is a class of small Gproteins found on GSVs and they possess intrinsic GTPase activity. Inhibition of AS160 suppresses Rab GTPase activity and therefore increases the production of GTP (guanosine triphosphate) which results in the activation of Rab **(8)** [33]. Rab regulates GSV trafficking and its activation is responsible for the translocation and fusion of the GSV with the cell membrane which results in the expression of GLUT4 on the cell surface **(9)** [20, 33].

### Insulin resistance

Insulin resistance (IR) is a characteristic pathophysiological feature of T2DM, and is defined as a state in which an abnormally higher amount of insulin is required to elicit a quantitatively normal biological response [34]. In other words, IR refers to the inability of an insulin-sensitive cell to respond to normal physiological concentrations of insulin, which results in an impairment of the insulin-induced glucose uptake and downstream metabolism by a cell. As tissues and organs become increasingly resistant to insulin, the pancreatic *β*-cells compensate by increasing insulin synthesis to maintain normal blood glucose levels. This leads to hyperinsulinemia; high plasma insulin levels. Chronic overstimulation of the *β*-cells, which can be due to overnutrition and/or insulin resistance, leads to *β*-cell exhaustion and dysregulation and ultimately causes *β*-cell failure and insulin deficiency. IR and insulin deficiency contributes to hyperglycaemia which progresses to glucose intolerance and diabetes [35].

Insulin resistance precedes T2DM by 10–20 years and is central to the development of the disease [30, 36]. Majority of patients with T2DM are insulin resistant. Attenuating IR lowers the risk of T2DM [36]. The exact aetiology of IR is unclear, however obesity; especially excess visceral fat, and physical inactivity are strongly linked to its development. Adipose tissue is a storage site for lipids and also secretes the adipocyte-derived secretory proteins collectively known as adipokines that affects key physiological process such as energy metabolism and inflammation [37]. Free fatty acids released during adipocyte lipolysis changes the adipokine secretory profile to one that leads to inflammation and insulin resistance [28, 38]. In addition, accumulation of free fatty acids around liver and skeletal muscles impedes insulin signalling and reduces insulin sensitivity [29, 39]. Skeletal muscle is the principal site of insulin-mediated glucose disposal following a meal. Therefore, muscle insulin resistance is considered the primary defect in T2DM [30]. Muscles metabolise a significant amount of glucose for energy and exercise increases glucose uptake by skeletal muscles [40]. Furthermore, the insulin de-sensitizing effects of ectopic lipid accumulation and a sedentary lifestyle also causes muscle insulin resistance [41].

## Current therapies for T2DM

Several pharmaceutical therapies are currently available for the treatment of T2DM (Table 1) with different modes of action. For example, sulfonylureas and meglitinides are insulin secretagogues; i.e. they stimulate insulin secretion by pancreatic *β*-cells [3]. Biguanides and thiazolidinediones are insulin-sensitisers (also termed insulinomimetics) [3, 42–44]. Acarbose inhibits *α*-glucosidase in the lumen of the small intestine and retards the digestion and absorption of dietary carbohydrates, thus alleviating postprandial hyperglycaemia [45].

A newer class of anti-diabetic therapies is based on the antihyperglycaemic activity of the incretin hormones glucagon-like peptide-1 (GLP-1), and glucose-dependant inhibitory peptide (GIP). GLP-1 and GIP regulates appetite and satiety, inhibits glucagon secretion and promotes insulin production. They are produced by enteroendocrine cells in the gastrointestinal tract and are rapidly degraded by dipeptidyl peptidase-4 (DPP-4). Analogues and receptor agonists of GLP-1 and GIP, and DPP-4 inhibitors are therefore therapeutic against T2DM [46–48].

Sodium glucose co-transporter (SGLT) inhibitors are another novel class of oral medications for treating diabetes [49]. SGLT-2 proteins in the renal proximal tubules are responsible for the reabsorption of glucose from the glomerular filtrate into the blood stream [49]. Selective inhibition of SGLT-2 facilitates the removal glucose in urine by inhibiting the reabsorption of glucose in the kidney [49–51]. SGLT-1 proteins are glucose transporters involved in the absorption of glucose in the small intestines and inhibiting SGLT-1 protein delays intestinal glucose absorption and lower postprandial blood glucose levels. SGLT-1 inhibitors are still in the research phase however a dual SGLT-1/2 inhibitor, sotagliflozin, which inhibits both SGLT-1 and SGLT-2 has shown efficacy in controlling blood glucose levels and represents a promising treatment for both T1DM and T2DM [52, 53].

**Table 1:** Common oral anti-hyperglycaemic therapies and their respective modes of action

Class	Example structure	Primary mode of action	Non-hypoglycaemic benefits	Side effects	Contraindications	References
Sulfonylureas, e.g. tolbutamide, glipizide, glyburide, glimepiride	Tolbutamide	Increase pancreatic insulin secretion	–	Hypoglycaemia Weight gain Increased risk of CVD	Renal and hepatic disease Predisposition to hypoglycaemia T1DM/pancreatic diabetes Pregnancy Major surgery Sulfa drug allergy	[3]
Meglitinides, e.g. repaglinide, nateglinide	Repaglinide	Increase pancreatic insulin secretion	–	As per sulfonylureas	As per sulfonylureas	[3]
Biguanides, e.g. metformin	Metformin	Increase insulin sensitivity Reduce hepatic glucose output	Does not cause weight gain Monotherapy does not cause hypoglycaemia Improves lipid profile and other vascular risk factors Anti-atherogenic	Gastrointestinal issues Metallic taste Possible impairment of vitamin B12 and B9 absorption Lactic acidosis Risk of hypoglycaemia in combination therapy	Renal and hepatic disease Cardiac or respiratory insufficiency History of lactic acidosis Severe infection Pregnancy Alcohol abuse	[3, 39, 40]
Thiazolidinediones (glitazones) e.g. pioglitazone, rosiglitazone	Rosiglitazone	Increase insulin sensitivity	Decrease blood pressure Anti-inflammatory activity Beneficial vascular effects	Hepatotoxicity Weight gain Fluid retention Congestive heart failure Bone fractures	T1DM Hepatic disease Class III or IV heart failure Pregnancy	[3, 39, 40]
*α*-Glucosidase inhibitors, e.g. acarbose	Acarbose	Reduce absorption of dietary carbohydrates	Low risk of hypoglycaemia Does not cause weight gain Protects again microvascular complications Potential to delay development of DM in pre-diabetics	Gastrointestinal disturbances: Flatulence Diarrhoea	Renal and hepatic disease Irritable bowel syndrome Pregnancy Lactation Children < 12 years	[41]
Incretin mimetics/GLP-1R agonists, e.g. exenatide, liraglutide	Exenatide	Delay gastric emptying	Low risk of hypoglycaemia Reduce appetite	Nausea Vomiting Diarrhoea	Severe renal impairment T1DM Pregnancy Lactation	[42, 44]
Incretin-enhancing DPP-4 inhibitors, e.g. sitagliptin, vildagliptin	Sitagliptin	Delay gastric emptying	Low risk of hypoglycaemia	Increased risk of infection Headache	History of hypersensitivity to sitagliptin	[42, 44]
SGLT-2 inhibitors, e.g. dapagliflozin, empagliflozin, ertugliflozin	Dapagliflozin	Reduce glucose reabsorption in the kidneys	Low risk of hypoglycaemia Reduces body weight Blood pressure reduction	Increased risk of urinary tract infections Risk of ketoacidosis	Renal disease	[49–51]
SGLT-1/2 co-inhibitors		Reduce glucose reabsorption in the kidneys Delay intestinal glucose absorption	Low risk of hypoglycaemia Reduces body weight Blood pressure reduction	Increased risk of urinary tract infections Risk of ketoacidosis Diarrhoea	Renal disease	[52, 53]

Currently, insulin secretagogues and sensitisers are the most widely prescribed classes of anti-diabetic therapies. The biguanide, metformin is the most common T2DM medication, and is prescribed as a first-line therapy for diabetes patients who are overweight or obese [54, 55]. Metformin increases insulin sensitivity by enhancing glucose uptake and utilisation by muscles and also suppresses gluconeogenesis in the liver [55, 56]. With the range of antihyperglycaemic agents available, medical practitioners can select an appropriate therapy or co-therapies based on the requirements of the patient while taking into consideration the side-effects of the various medications.

In patients with advanced T2DM, oral medications may fail to adequately control hyperglycaemia and diabetes symptoms. Such patients are placed on insulin replacement therapy. Insulin administration is usually via insulin pen injectors. Insulin pumps are also an option; however, these are traditionally only used by T1DM patients [57]. Insulin therapy is recommended when 2–3 months of dual oral therapy with anti-diabetic medications fail to achieve HbA1c levels ≤ 7% [58].

Although the conventional therapies for T2DM can assist in regulating blood glucose levels and manage the symptoms of diabetes, they are not without undesirable side effects. For example, insulin therapy and insulin secretagogues can cause a lethal reduction in blood glucose levels resulting in hypoglycaemia [3]. Sulfonylureas and thiazolidinediones can promote weight gain, while biguanides, *α*-glucosidase inhibitors and incretin axis-related therapies often cause adverse gastrointestinal effects (Table 1). Therefore, alternative and effective therapies with less harmful side effects are urgently required.

## Natural products to combat diabetes

Natural products are small molecules (secondary metabolites) originating from biological sources such as plants, animals or microorganisms of terrestrial or marine origin [59]. Secondary metabolites are not essential for the growth and development of the organism but are often unique to species offering structurally unique and selective bioactivities which make natural products an important source of leads for novel medicines [12, 60]. Modern strategies in natural product research are bioactivity-focused and involve the selection of species based on ethnopharmacological knowledge or traditional use, bioassay-guided isolation and identification of lead compounds, generation of NP libraries, and more recently, combining classical approaches with metabolomics to accelerate the dereplication process [61, 62]. In addition to the documented knowledge of ethnopharmacological or traditional use, plants can also be selected based on chemotaxonomical information, or plant families known to contain bioactive compounds of interest can also be chosen [59].

Plants that have been used for the treatment and management of diabetes in ethnomedicine, ranked in order of most widely cited include *Momordica charantia*, *Catharanthus roseus*, *Syzygium cumini*, *Trigonella foenum*-*graecum*, *Phyllanthus emblica*, *Phyllanthus niruri*, and *Morus alba* [13]. *M. charantia* (bitter melon) is used for treating diabetes in traditional medicine across Asia, regions of Africa, South America, the Caribbean, the Middle East and England [13]. It is the most widely cited plant with antidiabetic activity and perhaps best illustrates the importance of secondary metabolites in diabetes. The unripe fruit, aerial parts and powdered seeds from *M. charantia* have potent insulinomimetic and insulin secretagogue activity in animal models [63, 64], in vitro [65] and in clinical trials [66, 67]. The main secondary metabolites responsible for the anti-hyperglycaemic activity of *M. charantia* are charantin, momordicin, *p*-insulin and vicine [66, 68]. Charantin, a mixture of steroidal saponins from *M. charantia* fruit lowered blood glucose levels in rodent models of T2DM [69, 70], while the alkaloid momordicin promoted pancreatic *β*-cell regeneration [68]. Baldwa [71], and Khanna [72] observed evidence for the insulinomimetic properties of *p*-insulin by demonstrating that subcutaneous administration of *p*-insulin; a small, insulin-like polypeptide isolated from *M. charantia;* reduced blood glucose in patients with diabetes in a clinical setting. However, both these studies are limited by small participant numbers, and there have been no other studies on the effect of *p*-insulin in humans. A study by Yibchok-Anun [73] showed that a crude protein extract from *M. charantia* fruit increased plasma insulin concentrations, and significantly decreased plasma glucose levels in both normal, and diabetic rats. In addition, the authors discovered that the protein extract increased glucose uptake by myocytes and adipocytes in vitro [73]. These findings suggest that *M. charantia* contains insulin secretagogue peptides in addition to insulinomimetic *p*-insulin. This highlights the prudence of further study into the antidiabetic properties of *M. charantia,* and the role of *p*-insulin as a potential insulin replacement in patients with diabetes.

## Screening plants and natural products for antidiabetic activity

A wide range of in vivo and in vitro experimental models are used in T2DM research and for screening potential antidiabetic agents. The involvement of the pancreas in diabetes and the isolation of insulin were first achieved in pancreatectomised dogs [74–76]. The role of insulin resistance and obesity in the pathology of diabetes has been investigated in vivo using rodent models of rats, mice, rabbits [77], and in vitro using muscle cells [78, 79] and adipocytes [80]. Antidiabetic agents have been assessed for blood glucose lowering activity in rodent models, insulin secretagogue activity evaluated in isolated pancreatic *β*-cells [81], while muscle cells [82] and adipocytes have been used for screening insulin-like activity [83]. The complex, multi-organ nature of diabetes necessitates the use of multiple experimental models as no single model can accurately portray all the pathological aspects of the disease [84]. The availability of a range of experimental models makes it possible to select one that is appropriate for the aims of a study. The proceeding sections of this review will focus primarily on in vitro methods in diabetes research.

### In vivo animal models

Mice (*Mus musculus*) and rats (*Rattus norvegicus*) are the most common animal models in T2DM research [85]. These animals make suitable models as they share metabolic and signalling pathways with humans [86], and develop insulin resistance and hyperglycaemia similar to human T2DM [87]. In addition, the genetics and physiology of mouse and rat models are well understood and documented.

#### Obese mouse and rat models

There is strong evidence supporting a link between obesity and T2DM. Fluctuations in a number of adipokines including leptin, adiponectin, and resistin has been shown to play a role in insulin resistance and the development of diabetes [88]. Therefore, many rodent models have been modified to manifest obese conditions for research (Table 2). Obese models are a valuable approach for studying the interaction between obesity and T2DM because, in addition to hyperglycaemia and compensatory hyperinsulinemia, obese models also develop dyslipidaemia and other metabolic abnormalities associated with obesity [85].

**Table 2:** Obese mouse and rat models used in T2DM research

Model	Polygenic	Monogenic	References
Mouse			
db/db mouse		✓	[93]
KK (Kuo Kundo) mouse	✓		[86, 94]
KK A^Y^ (KK yellow obese) mouse	✓		[92]
M16 mouse	✓		[86, 95]
Nagoya-Shibata-Yasuda (NSY) mouse	✓		[96]
New Zealand Obese (NZO) mouse	✓		[86]
ob/ob mouse		✓	[93]
Tsumura Suzuki obese diabetes (TSOD) mouse	✓		[97, 98]
Rat			
JCR/LA-cp (James C Russell/LA corpulent) rat	✓		[86]
Otsuka Long-Evans Tokushima Fat (OLETF) rat	✓		[90]
Spontaneously hypertensive rat/NIH-corpulent (SHR/N-cp) rat		✓	[86]
Zucker Diabetic Fatty (ZDF) rat		✓	[86]
Zucker fa/fa rat		✓	[86]
Zucker Fatty Diabetes Mellitus (ZFDM) rat		✓	[99]

In diabetes research, polygenic animal models are more relevant than monogenic models as most cases of T2DM in humans are polygenic in nature. These models are characterised by insulin resistance, glucose intolerance, hyperglycaemia and hyperlipidaemia [89–91]. Except for the Kuo Kondo yellow obese (KK A^y^) mouse, all these models also exhibit hyperinsulinemia. The KK A^y^ either produces very little insulin, or no insulin at all [91].

#### Non-obese mouse and rat models

Non-obese animal models (Table 3) are also important in diabetes research as a significant proportion of T2DM patients are not obese. The distinguishing feature of non-obese diabetics is reduced insulin secretion as opposed to the insulin resistance observed in obese diabetes [99].

**Table 3:** Examples of non-obese animal models of T2DM

Animal	Model	References
Mouse	TallyHo/Jng mouse	[91]
	Non-obese C57BL/6 (Akita) mutant mouse	[86]
	ALS (alloxan sensitive)/Lt mouse	[86]
Rat	Goto-Kakizaki (GK) rat	[101]
	Cohen diabetic rat	[102]
	Torri rat	[103]

The Goto-Kakizaki (GK) rat is one of the best characterised polygenic, non-obese models of T2DM [100]. The GK rat is born with low numbers of islets and by the time the rats reach adulthood, they display a 60% reduction in *β*-cell mass. They also do not develop hyperglycaemia until around 3–4 weeks of age and maintain a normal BGL unless challenged with glucose. The early loss of pancreatic *β*-cell mass in these animals means they are perhaps better suited for T1DM research and has a limited capacity for accurately representing T2DM [85].

The non-obese C57BL/6 mutant mouse is a common mouse model of non-obese diabetes. This model exhibits polydipsia and polyuria, and the gradual loss of *β*-cell mass caused by a spontaneous mutation in the INS2 gene results in progressive hypoinsulinemia leading to hyperglycaemia [85]. Similar to the GK rat, the loss of functional *β*-cell makes this model suitable for T1DM research and is currently used primarily for islet transplantation studies [103, 104].

#### Animal models with diet-induced T2DM

Human T2DM is predominantly associated with high caloric intake and energy imbalance. Consequently, animal models with diabetes caused via nutritional manipulation (Table 4) are especially relevant systems for investigating how diet influences the development of T2DM, nutritional intervention research and the effect of dietary phytochemicals in the prevention and management of T2DM [85, 105]. Traditionally, mice or rats fed on a high-fat diet have been the models of choice. However, other animal models developed in recent years are worth consideration as presented below.

**Table 4:** Examples of animal models with diet-induced diabetes

Animal	Model	References
Mouse (*Mus musculus*)	High-fat-fed C57/BL 6 J mouse	[86]
Rat (*Rattu*s spp.)	High-fat-fed rat	[106–108]
Other rodent	Israeli sand rat/Desert gerbil (*Psammomys obesus*)	[109]
	Spiny mouse (*Acomys cahirinus*)	[109]
Fish	Diet-induced obese zebra fish (*Danio rerio*)	[110]
Insect	High-sugar diet (HSD) fruit fly (*Drosophila melanogaster*)	[111, 112]

#### Chemically-induced models of diabetes

**Fig. 6:**
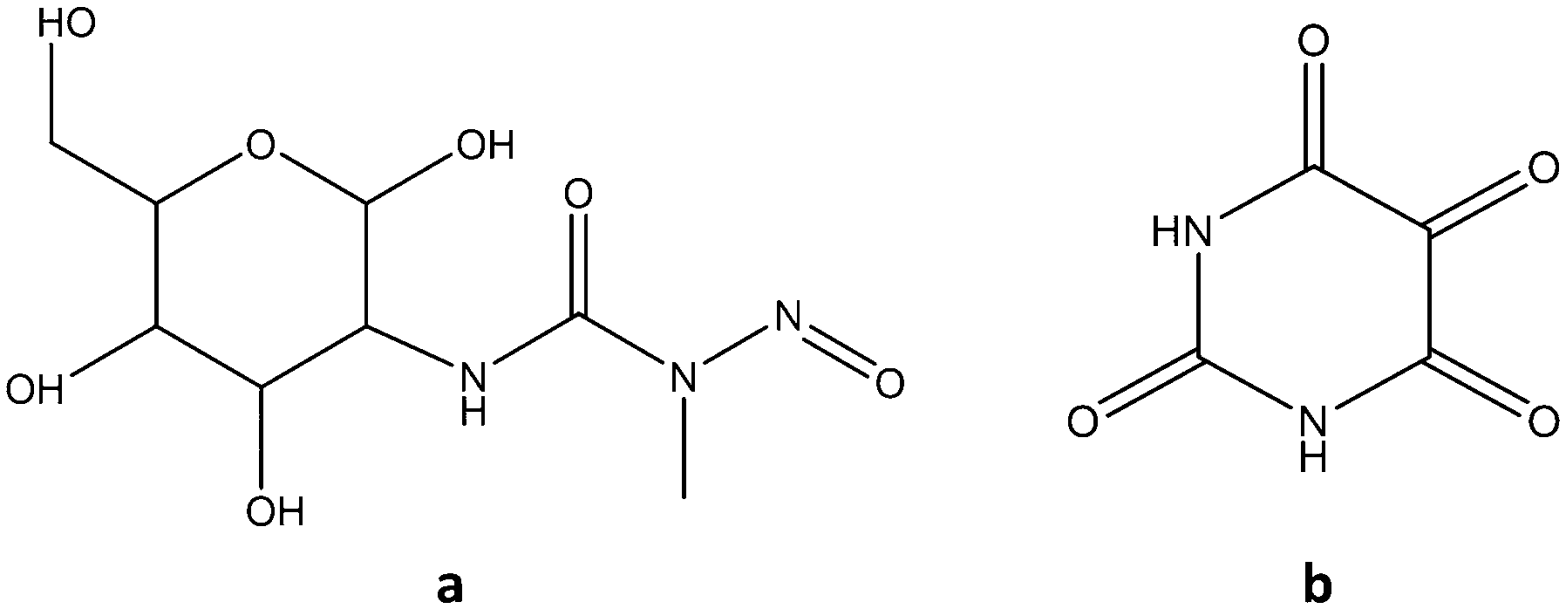
Structures of **a** streptozotocin, and **b** alloxan

Animal models with chemically-induced diabetes are the most frequently used models for screening plants and natural products for antihyperglycaemic activity. Streptozotocin (STZ) and alloxan (ALX) (Fig. 6) are the most common diabetogenic chemicals administered for inducing experimental diabetes [87]. Both these compounds are preferentially taken up by the GLUT2 transporter in pancreatic *β*-cells. The accumulation of these chemicals leads to cytotoxic effects in the *β*-cells resulting in cell death [112].

Streptozotocin causes necrotic *β*-cell death via DNA alkylation [112]. The loss of *β*-cells means that STZ-induced diabetes resembles T1DM. However, it is still a relevant model for investigating *β*-cell-protective effects and antihyperglycaemic agents. A model with reduced *β*-cell mass can be created by administering STZ to neonatal rats. This is a useful model for studying agents that may aid *β*-cell regeneration [90]. The structural, functional and biochemical changes observed in STZ-induced diabetic animals are similar to those observed in humans, making this a clinically-relevant model for studying the various aspects of diabetes. However, STZ also causes toxic effects on the liver and kidneys. In addition, the level of severity of diabetes induced by STZ is highly variable and has low reproducibility as many factors (e.g. route of administration, animal species, body weight and diet) influences the activity of STZ [113].

Diabetes induced by ALX is insulin-dependent [87]. ALX produces reactive oxygen species (ROS) in the presence of thiols which results in the necrotic death of *β*-cells. This makes ALX-induced diabetic animal models useful for the study of ROS-mediated cytotoxicity in *β*-cells. Inhibition of glucokinase by alloxan inhibits glucose-stimulated insulin secretion by pancreatic *β*-cells. Therefore, it is suitable for investigating antidiabetic agents where the mechanism of action is independent of the activity of *β*-cells [112].

Despite their ability to produce diabetic conditions in animals, the effects of these compounds cannot reproduce all the characteristics of human diabetes. Furthermore, the nephrotoxic and hepatotoxic effects of ALX and STZ limits their usefulness. Antioxidants affect the effectiveness of ALX as a diabetogenic agent. This should be taken into account when using an ALX-induced diabetic animal model for the evaluation of natural products with antioxidant activity [87]. Streptozotocin is preferable over alloxan as it is significantly more stable, has a longer half-life and generates less ROS [112].

In vivo testing methods have several shortcomings. It is often challenging to establish the mechanism of action of a test substance or intervention as the end-result (e.g. reduction in blood glucose) is often the result of complex interactions between different tissues and organ systems in a living animal. In addition, large numbers of animals are needed to achieve results with adequate statistical power. Animal husbandry requires food, medications, special animal housing facilities and personnel specially trained in animal handling. Larger quantities of the test substance are required to administer to the animals which is particularly inconvenient given that the isolation and purification of natural products is often time consuming and expensive process that produce low yields of the compound of interest [81, 114]. In vitro testing methods, such as those discussed in the next section, provide an alternative that overcomes these shortcomings.

### In vitro models

Glycaemic control; the control of blood glucose levels, is the primary goal of antidiabetic therapy [3]. Therefore, the search for antidiabetic agents focuses on the various targets and mechanisms by which blood glucose levels can be lowered to normal levels (euglycaemia or normoglycaemia; fasting plasma glucose < 7.0 mmol/L [115]). Antihyperglycaemic agents function via five main mechanisms that can be evaluated via in vitro techniques as presented in Table 5. In vitro testing is particularly useful for mechanistic-based screening of plant extracts and pure natural products [116] and is recommended as the first step towards screening potential antidiabetic agents [13, 117]. In vitro techniques can be employed to randomly screen selected plants or plants selected based on ethnopharmacological knowledge for potential antidiabetic activity. They can also be used for supporting antidiabetic activity observed in human or animal studies through elucidation of mechanism(s) and mode(s) of action.

**Table 5:** Key mechanisms of antihyperglycaemic agents

Mechanism	Function	References
Delay gastric emptying	Slows the passage of food from the stomach into the small intestine thereby slowing the absorption of glucose Usually achieved via the activation of the GLP1 receptor, or the inhibition of DPP-4	[118, 119]
Enzyme inhibition	Inhibition of carbohydrate digesting enzymes (e.g. alpha-amylase and alpha-glucosidase) slows release of glucose from digested food Inhibition of enzymes involved in hepatic glucose metabolism (e.g. glucose-6-phosphatase) or reduces hepatic glucose output	[120–122]
Insulin secretagogue activity	Stimulates insulin secretion from pancreatic β-cells	[82]
Insulin-like/insulin sensitizing activity	Enhances glucose uptake by target tissue by stimulating the cell surface expression of GLUT transporters (liver, adipose tissue, and skeletal muscle)	[84]
Reduce intestinal glucose uptake	Reduce absorption of glucose from digested food and into the blood stream either through direct inhibition of enterocyte SGLT-1 or GLUT-2 transporter proteins	[123]
Reduce glucose reabsorption in the kidney	Increases the amount of glucose excreted in urine by inhibiting SGLT-2 proteins in the kidneys which prevents the reabsorption of glucose into the blood stream	[50–52]

Screening for insulin secretagogue activity, insulin sensitizing potential and enzyme inhibition are popular approaches in current diabetes research as these can be studied using cell lines or biochemical assays without the use of animals, are therefore cheaper, more efficient, does not require stringent ethics protocols and can be easily adapted for high-throughput screening.

#### Insulin-secretagogue activity

Plant extracts or isolated natural products that potentiate glucose-stimulated insulin secretion by pancreatic *β*-cells can be investigated using several in vitro models such as perfused pancreas, isolated islets, and various *β*-cell lines. For instance, a perfused rat pancreas model [123] and the INS-1 pancreatic *β*-cell line [124] were used to investigate the effect *Orthosiphon aristatus* leaf extract on insulin secretion. Both studies reported that *O. aristatus* leaf extract stimulated glucose-induced insulin release in the perfused pancreas and in vitro [123, 124]. Ahangarpour [125] showed that the leaf extract from *Morus nigra* produced a significant increase in insulin production in isolated mouse islets. Hannan and co-authors established the insulin secretagogue activity of *Ocimum sanctum* leaves [126] and *Asparagus racemosus* roots [127] using all three models demonstrating the compatibility of these models for screening potential insulin secretagogues from plants.

##### Pancreatic primary cells

Insulin secretagogue activity can be assessed either with primary cells or immortalised cell lines. Primary cells are isolated via enzyme digestion of pancreatic tissue, are therefore genetically and phenotypically identical to their parent tissue, and conserve metabolic process and biochemical markers seen in vivo [128]. However, primary cells are short-lived and it is usually difficult to obtain a pure population of primary cells [128]. Variability between cells obtained from different individual animals impacts the reproducibility of results. In addition, a regular supply of fresh tissue from animals must be maintained to provide cells for in vitro experiments [129]. Therefore, despite the advantages of primary cells, their use is limited by tissue availability.

##### Established pancreatic cell lines

Several immortalised insulin-secreting cell lines are available commercially. Examples of insulin-secreting cell lines frequently encountered in diabetes research include RIN, HIT-T15, *β*-TC and MIN6 [130] some of which are described below.

INS-1: The INS-1 cell line is a genetically modified cell line established from an x-ray-induced rat insulinoma [131] and is one of the most physiologically relevant *β*-cell models currently available for studying *β*-cell function. A study by Lee and co-authors [124] showed that a non-polar extract prepared from the leaves of *Orthosiphon stamineus* (also known as *O. aristatus*) stimulated insulin production in INS-1 *β*-cells via the activation of PI-3K and Akt, and protected the cells against glucotoxicity. An extract from *Angelica dahurica* tested on the INS-1 cell line exhibited insulinotropic activity by enhancing the activation of GPR119 (G protein-coupled receptor 119); a receptor involved in the glucose-stimulated insulin secretion in pancreatic *β*-cells [132]. Unlike many other insulin-secreting cell lines, INS-1 is responsive to glucose at physiological concentrations [131, 133] making it a valuable approach for evaluating the effect of test substances on glucose-stimulated insulin production. In addition, INS-1 cells are proliferative and have been passaged up to 80 times without any substantial changes in morphology or function [131]. However, these cells produce low amounts of insulin and must be cultured with 2-mercaptoethanol to sustain growth [129, 131]. Although 2-mercaptoethanol assists INS-1 cell survival by increasing intracellular glutathione (GSH) levels, high GSH levels are associated with reduced insulin production [134].

HIT-T15: This HIT cell line is a hamster insulinoma cell line established by transforming Syrian hamster *β*-cells with SV40 (simian virus 40) [135]. Out of the four sub-clones, only HIT-T15 is responsive to glucose stimulation [129, 135]. Rotshteyn and Zitho [81] used HIT-T15 cells to evaluate the sulfonylurea-like activity of *Momordica charantia* and *Panax quinquefolius* (American ginseng) using glibenclamide as a control. The HIT-T15 cells were a suitable clone for screening plant extracts for sulfonylurea-like activity via insulin secretion and sulfonylurea receptor 1 (SUR1)-binding studies [81]. In another study, HIT-T15 cells treated with whole plant extracts of *Corydalis edulis* displayed enhanced glucose-independent insulin secretion [136].

MIN6: MIN6 is a mouse insulinoma cell line produced by SV40 transfection [137]. This cell line expresses GLUT2, exhibits a glucose-response similar to normal *β*-cells and maintains morphological and physiological characteristics of primary mouse *β*-cells [137, 138]. Glucose-stimulated insulin secretion is impaired in high passage MIN6 cells demonstrating that high passage numbers should be avoided [139]. The MIN6 cell line has been used to investigate the insulin secretagogue activity of saponins isolated from fruits of *M. charantia* [65]; and leaves of *Costus pictus* [140], *Geranium graveolens, Sarcopoterium spinosum,* and *Varthemia iphionoides* [141].

Immortalised cell lines tackle some of the issues common in primary cells. For example, immortalised cell lines can be cultured for extended periods and are proliferative. Cell populations should be homogeneous which improves the reproducibility of experiment results [84]. Nevertheless, immortalised cell lines have their own limitations. For instance, the replicative senescence of these cell lines has been muted with assorted transformation techniques, some of which include irradiation and viral infection. Consequently, these cell lines are essentially derived from tumours and have some form of genetic mutation [129]. Therefore, although these cell lines have been developed to preserve the genotype, tissue markers and insulin secreting activity to reflect those of the originating tissue as closely as possible, they are not functionally equivalent to primary cells. An issue that is particularly problematic in insulin-secreting cell lines is that some (e.g. In-111) are not responsive to glucose at physiological concentrations, while others (e.g. RINm clone) do not respond to glucose at all [129]. These limitations must be taken into consideration when using immortalised cell lines in diabetes research.

##### Measuring in vitro insulin production

Measurement of insulin production by pancreatic *β*-cells is a necessary step in studies designed to screen potential insulin secretagogues. Currently, radioimmunoassay and ELISA (enzyme-linked immunosorbent assay) are the most commonly used methods for the determination of insulin production in vitro [34, 124, 142–144].

#### Insulin-like/insulin sensitizing activity

The three main targets of insulin; the liver, muscles and adipose tissue; are also the major sites of insulin resistance in the body. Therefore, insulinomimetic and/or insulin sensitizing properties of compounds are screened using these tissues.

##### Liver

Insulin has several antihyperglycaemic actions on hepatocytes. Insulin stimulates glycogenesis by activating glycogen synthase and suppresses gluconeogenesis by inhibiting the gene expression of phosphoenolpyruvate carboxylase (the enzyme that catalyses the rate-limiting step in gluconeogenesis) and the final enzyme in gluconeogenesis; glucose-6-phosphatase [22]. Insulin also reduces gluconeogenesis indirectly by inhibiting lipolysis in visceral fat which reduces free fatty acids available for conversion as precursors into glucose in the liver [28]. Therefore, substances with potential insulinomimetic properties can be screened by evaluating their effects on these enzymes. In addition, even though not insulin-dependent, stimulating glucose uptake by hepatocytes via the GLUT2 transporter is another important hepatic hypoglycaemic mechanism of interest in diabetes [26, 145].

Models of hepatocyte function used in diabetes research include perfused whole livers, liver slices, primary hepatocyte suspensions or monolayers, and hepatocyte cell lines [84]. Perfused livers and liver slices exhibit the strongest similarity to in vivo hepatic metabolism, but they are not readily available (tissue is acquired from animals) and are only viable for a short time [84]. A comparable alternative is to use primary hepatocytes. For example, Zheng [146] tested the effect of *Entada phaseoloides* (a plant belonging to the legume family) seed extract and found that it reduced glucose production and decreased G6Pase activity. Although primary hepatocytes retain much of the original functional properties and gene expression profile of in vitro hepatocytes making them the closest model to in vivo liver function [147, 148] and is therefore a reliable alternative to using perfused livers and liver slices, the use of primary hepatocytes poses issues of high variability, low proliferation rates, high cost and limited availability [149].

Immortalised liver cell lines have become the model of choice in recent years and have been used in many studies to investigate the effects of natural products on glucose metabolism in hepatocytes. Examples of hepatic cell lines used in diabetes research include the human hepatocellular carcinoma cell lines HepG2 and Hep3B, and H4IIE and FTO-2B rat hepatoma cell lines [145, 150, 151]. The HepG2 cell line appears to be the most popular and has been used in multiple studies. For example, HepG2 cells were used to study the antidiabetic properties of *Morus alba* (white mulberry) fruit anthocyanins [152], *Perilla frutescens* sprout extract [153], *Sambucus nigra* (elderflower) flower extract [154], *Helicteres angustifolia* root extract [145], and *Brachylaena elliptica* leaf extract [155]. In HepG2, *M. alba,* and *P. frutescens* extracts suppressed gluconeogenesis by downregulating PEPCK and G6Pase activity, and *S. nigra*, *H. angustifolia* and *B. elliptica* extracts enhanced glucose uptake [145, 152–155].

##### Muscle

Muscle tissue accounts for approximately 80% of glucose uptake following a meal and is the main site of insulin-stimulated glucose disposal in the post-prandial state [30]. Insulin stimulates the translocation of the GLUT4 transporter from intracellular storage and on to the cell membrane facilitating glucose uptake by the tissue. The insulin-stimulated translocation of the GLUT4 transporter is reduced in insulin resistance and diabetes [156]. Therefore, the effect of test substances on GLUT4 translocation and glucose uptake in muscle tissue are useful for screening substances with potential insulinomimetic activity.

Insulinomimetic properties of plant extracts have been studied using animal hemi-diaphragms, human primary muscle cells and immortalised muscle cell lines. Isolated rodent hemi-diaphragms were used to successfully evaluate the beneficial effects of extracts from *Barleria noctiflora* [157], *Cocculus orbiculatus, Leea indica, Ventilago maderaspatana* [158], and of functional beverages containing *Orthosiphon aristatus* (‘cat’s whiskers’ plant) [159], on muscle glucose uptake and utilisation. All extracts promoted glucose uptake by the hemi-diaphragm muscle tissue, while *C. orbiculatus, L. indica,* and *V. maderaspatana* also increased glycogen storage. Another model, myotubes differentiated from isolated human satellite cells have been used to study the effect of elderberries on glucose and fatty acid uptake. There was an increased uptake of glucose and oleic acid in muscle cells treated with the elderberry extract [154, 160].

Among the immortalised cell lines, the C2C12 mouse myoblast cell line is a commonly used model for studies on T2DM and insulin resistance. For example, *Cassia abbreviate* [161] and *Helicteres angustifolia* root extracts [145, 161], *Ficus lutea* leaf extract [151] demonstrated enhancement of muscle glucose uptake in C2C12 cells. The upregulated expression of GLUT1 and increased translocation of GLUT4 were responsible for the increased glucose uptake in *C. abbreviate* [151]. The C2C12 cell line has been used to assist bioassay guided fractionation and preparation of plant extracts. For instance, Hetta [150] tested *Eruca sativa* (rocket) leaf extracts prepared with solvents of different polarities and found that the *n*-hexane-soluble fraction of a 95% ethanol extract and its fatty acid-rich fraction promoted glucose uptake in C2C12 cells.

##### Adipose tissue

The stimulation of lipogenesis, suppression of lipolysis, increase in GLUT4 translocation, and the resultant increase in cellular glucose uptake are actions of insulin that can be investigated using adipose tissue. Primary adipocytes isolated from rat epididymal adipose tissue, and differentiated 3T3-L1 mouse fibroblast cell lines are commonly used experimental models for screening antidiabetic activity [84]. As with all primary cells, the high variability, low proliferation rates, high cost and issues with availability of fresh tissue limits the use of primary adipocytes [149]. Therefore, immortalised cell lines such as the 3T3-L1 fibroblasts are preferable. The 3T3-L1 cell line was suitable for studying plant extracts promoting glucose uptake, inhibiting lipid accumulation, improving insulin sensitivity, and for bioassay-guided fractionation of crude extracts. For example, *Ipomoea batatas* (sweet potato) leaf extracts and extract fractions enhanced glucose uptake by 3T3-L1 adipocytes [162]. Leaf extracts from *Cudrania tricuspidata* inhibited lipid accumulation in differentiated 3T3-L1 cells [77], whilst leaf extracts from the plant, *Eruca sativa* exhibited an adipogenic activity comparable to the thiazolidinedione antidiabetic drug, rosiglitazone [150]. In addition, the leaves from *Eugenia polyantha* potentiated insulin action and improved insulin sensitivity by promoting expression of CEBP-alpha, GLUT4, and adiponectin in this cell line [80]. Therefore, the 3T3-L1 cell line is an important model for determining the antidiabetic mechanisms and efficacy of plant extracts.

#### Enzyme inhibitors

There are a number of enzymes of interest in diabetes therapy which can be targeted for investigation. These enzymes have a variety of substrates, however they all influence blood glucose levels to normality (Table 6). As a result, the inhibition of these enzymes leads to a reduction of blood glucose; the primary goal in antidiabetic therapy [163–166].

**Table 6:** Enzyme targets for antidiabetic therapy

Enzyme	Function	Effect of inhibition	References
*α*-Amylase	Hydrolysis of starch to oligosaccharides in the mouth and small intestine	Delays carbohydrate digestion → slower absorption of glucose from small intestine → reduces postprandial hyperglycaemia	[165, 168]
*α*-Glucosidase	Hydrolysis of oligosaccharides and sucrose to glucose in the small intestine	Delays carbohydrate digestion → slower absorption of glucose from small intestine → reduces postprandial hyperglycaemia	[165, 168]
Glucose-6-phosphatase	Dephosphorylation of glucose-6-phosphate to produce glucose (rate-limiting step in gluconeogenesis)	Prevents the increase of BGLs during the fasted state by inhibiting gluconeogenic glucose synthesis	[166]
PTP-1B (protein tyrosine phosphatase-1B)	Dephosphorylation of insulin receptor, and insulin receptor substrates 1 and 2	Enhances insulin receptor and IRS-1/IRS-2 phosphorylation → increase translocation of glucose transporters for glucose uptake → reduce BGL	[167]
DPP-4 (dipeptidyl peptidase-4)	Breakdown of GLP-1	Increases the level of endogenous GLP-1 → lowers production of glucagon and increases insulin production → lowering of BGLs postprandially	[47, 167, 169]

In recent years, the inhibition of *α*-amylase and *α*-glucosidase has been the most common assays used for screening large numbers of medicinal plants. Both *α*-amylase and *α*-glucosidase are carbohydrases involved in the digestion of carbohydrates to produce glucose. There are two forms of *α*-amylase: salivary/parotid *α*-amylase, and pancreatic *α*-amylase [169]. Both forms hydrolyse starch into oligosaccharides. The intestinal brush border enzyme, *α*-glucosidase breaks down oligosaccharides into glucose, and sucrose into glucose and fructose. Inhibitors of *α*-glucosidase are highly valuable as antihyperglycaemic agents because they inhibit the hydrolysis both of starch and sucrose [167]. A review of literature reveals a number of approaches for screening plant extracts for *α*-amylase and *α*-glucosidase inhibitory activity as summarised below.

##### Colorimetry

**Fig. 7:**
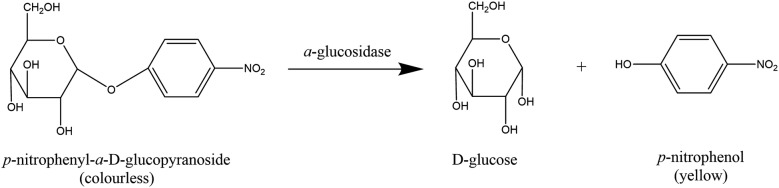
Hydrolysis of *p*-nitrophenyl-*α*-d-glucopyranoside to *p*-nitrophenyl by *α*-glucosidase (Adapted from [176])

Both the activity of *α*-amylase and *α*-glucosidase can be monitored by colorimetric methods. A colorimetric assay based on the colour change of dinitrosalicylic acid has been used to screen various medicinal plants [170–172], herbs and spices [121, 173]. The *α*-glucosidase inhibition assay uses *p*-nitrophenyl-*α*-d-glucopyranoside (PNPG) as the substrate. PNPG is a colourless molecule, which produces a yellow coloured product upon hydrolysis by *α*-glucosidase (Fig. 7). Numerous studies have utilised this method for screening medicinal plants and natural products for *α*-glucosidase inhibitors [121, 174, 175].

##### Fluorimetry

A fluorescence-based biochemical assay for determining enzyme activity is available for *α*-amylase but not for *α*-glucosidase. The substrate used in the assay is a modified, starch-derived molecule labelled with a special dye. Enzymatic hydrolysis of the substrate produces fluorescent fragments. Enzyme activity is proportional to fluorescence intensity which can be measured with a fluorometer or fluorescence microplate reader [177]. This assay is highly sensitive and convenient for fast screening of potential enzyme inhibitors. However, at present, it is not widely used perhaps due to the high cost and low stability of the substrate. Using the fluorescence-based assay, Jhong [178] established that curcumin, berberine, catechin, and quercetin were more powerful *α*-amylase-inhibitors than the antidiabetic drug acarbose, with curcumin showing the highest level of inhibitory activity (7.7 fold greater inhibition compared to acarbose). Yilmazer-Musa [179] utilized a similar method to demonstrate that green tea, white tea and grape seed extracts were good inhibitors of *α*-amylase.

##### Molecular docking

Computer-aided molecular docking is a novel approach for screening enzyme inhibitors and receptor agonists in diabetes research. Specialised software uses high resolution, three-dimensional molecular models of the target protein (e.g. receptor or enzyme) as a template to predict novel ligands that can bind to, and potentially modulate its activity [180]. The template structure is acquired via homology modelling or crystallography. Docking algorithms ‘*pose*’ molecules within the target active site in different orientations and conformational degrees of freedom in rapid succession which allows the software to screen compound databases in a high-throughput manner. Integrated scoring functions predict bioactivity based on interactions between the ligand and target protein taking into account factors such as ligand shape, electrostatic compatibility with the target, solvation effects, binding energies, and enthalpy and entropic effects; all of which are important determinants of successful receptor-ligand interaction [181].

Common therapeutic targets in diabetes investigated via virtual screening are *α*-glucosidase and *α*-amylase. The first computational scavenging for *α*-glucosidase inhibitors screened 85,000 compounds and identified 13 novel inhibitors [120]. Later studies by others screened 47 compounds and identified 10 inhibitors for *α*-amylase and *α*-glucosidase including curcumin, berberine, catechin, quercetin and rutin [178].

**Fig. 8:**
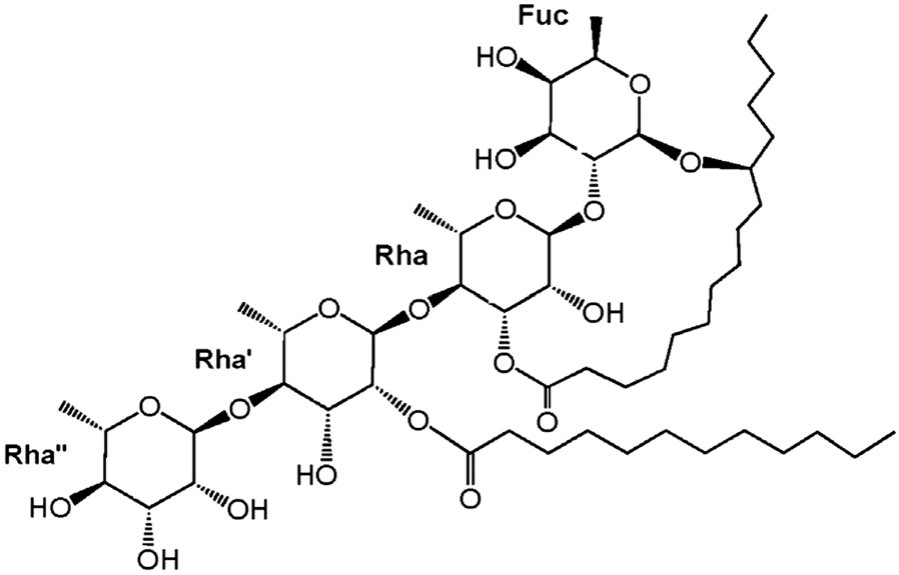
Chemical structure of pescaprein V

Molecular docking can also be used to predict bioactivity of compounds identified from plant extracts which enable further characterisation and development of bioactive compounds/mixtures with better efficacy. For example, Rosas-Ramirez [182] discovered the *α*-glucosidase inhibitory potential of four resin glycosides; pescaprein I, pescaprein V, purginoside II and purgin III, found in the morning glory family of plants (Convulvulaceae). The investigators docked the resin glycosides against MAL12, a yeast *α*-glucosidase involved in maltose and sucrose catabolism. Pescaprein V (Fig. 8) was the most potent inhibitor with a K_i_ (theoretical inhibition constant) of 1.49 µM [182].

The OH-3 of the fucose unit of pescaprein V is capable of hydrogen bonding with the NH (bond length 1.796 Å) or CO (bond length 2.174 Å) groups of the HIS279 residue in the catalytic site. Pescaprein V binds to *α*-glucosidase with high affinity. The binding conformation and orientation of pescaprein V, which is similar to that of acarbose, causes steric impedance which restricts the access of dietary carbohydrate substrates to the catalytic pocket thereby inhibiting enzymatic cleavage [182].

Literature published over recent years on studies such as the above shows that molecular docking is a powerful tool that can screen large libraries of small molecule candidates quickly and inexpensively. However, it requires specialised software, technical expertise and is only useful for virtual screening compounds where the structure is already known.

## Metabolite profiling

Bioactivity-based screening of plant crude extracts is one of the initial steps in the discovery of NPs with potential therapeutic use. Once a crude plant extract screens positive for a selected bioactivity—for example, the ability to reduce blood glucose levels—the next step is typically the bioactivity-guided fractionation of the crude extract, and the determination of their chemical ‘composition’ profiles. This information is important for identifying the NP(s) responsible for the observed bioactivity, let it be known or novel compounds. Typically, in a classical natural products approach the target compound(s) of interest needs to be isolated, purified and gross chemical structures characterised using analytical spectroscopic techniques [183, 184].

Undoubtedly, the isolation and successful purification of NPs is labour- and time-intensive; yet, it is a crucial process in NP research. The initial fractionation step generally involves a liquid–liquid extraction with an organic solvent such as ethyl acetate or chloroform and/or water depending on the targeted compound(s) polarity. These fractions are re-assayed for bioactivity, followed by further fractionation using chromatographic separation techniques [185]. The choice of separation technique is largely dependent on the solubility, volatility and stability of the molecules being separated [183]. Chromatographic separation techniques such as planar chromatography, low- or medium-pressure column chromatography, high-speed counter current chromatography, preparative high performance liquid chromatography; ion-exchange methods; solvent partitioning and crystallisation methods are common techniques utilized for the successful isolation and purification of natural products [186]. Following isolation and purification, structure elucidation of molecules is predominantly accomplished using nuclear magnetic resonance (NMR) spectroscopy and tandem mass spectrometry (MS^n^) experiments [187, 188].

Ultimately, bioactivity-guided fractionation can lead to the successful identification of novel bioactive NPs. However, this process cannot discriminate between novel and known NPs and can often result in the re-isolation of known NPs with already documented bioactivity which is an unproductive use of resources [185]. This workflow can be avoided by adopting a metabolite profiling approach where known bioactive compounds are differentiated from novel bioactive compounds early in the workflow by correlating bioassay results with the metabolite profile of an extract [185, 189]. This process of quick identification and elimination of known or undesirable compounds is referred to as dereplication. Dereplication is a vital step in NP discovery which prevents misapplication of resources to re-discover already documented/known compounds and ensures resources are focused on the more promising lead compounds [187].

### Metabolomics and metabolite profiling

Metabolomics is a well-established multidisciplinary field of analytical biochemistry that combines classical analytical tools with multivariate statistical analyses for the high-throughput identification, semi- and/or quantification of low molecular weight (< 1500 Da) metabolites in biological systems [190, 191]. The “*metabolome*” refers to the complement of endogenous small molecules (primary or secondary metabolites) present in a cell or organism, and is a direct representation of its physiological state and phenotype [191, 192]. Studying the metabolome enables researchers to observe interactions between the environment, the genome, and metabolism, detect perturbations in metabolic pathways in a diseased state, and to identify unique metabolites which are family, genus or species-specific. This is the primary goal of metabolomics. On the other hand, *metabolite profiling* targets the identification of a class of compounds, or metabolically-related compounds in a sample. Metabolite profiling can be *targeted*: i.e. the analysis of a specific compound or class of interest (e.g. flavonoids). Targeted analysis is particularly useful for applications requiring high sensitivity. For example, the monitoring of changes in phytohormones during plant growth or in response to stress requires high sensitivity as phytohormones are present in very minute concentrations [193–195]. *Untargeted* analysis refers to the simultaneous analysis and identification of multiple compounds in an NP extract, and this is a useful approach when the metabolite(s) of interest are unknown such as in the case of many NP discovery programs [196].

The metabolomics, in particular the untargeted chemical profiling workflow consists of experimental design, sample preparation and appropriate metabolite extraction, instrumental analysis [i.e. gas chromatography–mass spectrometry (GC–MS), liquid chromatography–mass spectrometry (LC–MS) or NMR] data acquisition, data processing, metabolite identification, and bioinformatic and statistical analysis [61, 197]. This is beyond the scope of this review and we would like to direct the reader to a number of key reviews [61, 188, 196, 197].

#### Sample preparation

Sample preparation is critical as it can impact the downstream quality and accuracy of both qualitative and quantitative results of subsequent analyses. The most appropriate sample preparation approaches are based on the analytical methods of choice, and in particular, the metabolite(s) of interest [198]. Firstly, plant samples are harvested and immediately frozen or treated with solvents to quench metabolism to alleviate any post-harvest changes to the metabolome [198]. Alternatively, plant samples may also be desiccated through freeze-drying, sun-drying, oven-drying, trap-drying, or using a rotary heating dryer but one should keep in mind that some of these processes could essentially lead to a loss of volatile or semi-volatile compounds during the process [199]. Freeze-drying is generally the most preferred method as the low temperature preserves the chemical integrity of the sample by minimising oxidation, thermal reactions and enzymatic degradation of metabolites [198, 199]. The dried samples are subsequently homogenized and extracted with suitable solvents. Solvent selection is also an important factor especially when targeting a specific class of metabolites as plant extracts contain complex mixtures of compounds with large structural diversity, polarity and solubilities [200]. It is often necessary to use a combination of polar (e.g. water, ethanol) and non-polar (e.g. chloroform, dichloromethane) solvents for the extraction process to ensure that a wide range of metabolites are extracted; a process typically referred to as untargeted chemical profiling. Selection of the appropriate extraction solvent(s) must take into consideration their toxicity, solubilisation index, selectivity, chemical reactivity, and pH [201, 202]. Depending on the experimental aims and the target metabolites, the volume of solvent, solvent:sample ratio, and the duration and temperature of extraction are other important factors which may require optimisation [202].

In most instances, the final step before instrumental analysis is filtration of the extract to remove particulate matter (e.g. carbohydrates and/or proteins) which may block the HPLC or MS system and can typically cause inhomogeneities when generating 1D-NMR data [198]. However, sometimes further ‘clean-up’ of the sample is required in order to eliminate or minimise biological matrix effects, and to remove compounds which may cause interference with the analysis or optimal functioning of analytical instruments. Matrix effects are particularly problematic in the case of complex biological samples such as blood, plasma, and tissue, and these often require a clean-up step prior to analysis [203]. Some frequently used clean-up methods include liquid–liquid extraction (LLE), solid phase extraction (SPE), supercritical fluid extraction (SFE), and protein precipitation (PPT), as presented below.

##### Liquid–liquid extraction

Liquid–liquid extraction is a classic separation technique involving the partitioning of the sample between two immiscible solvents; usually water and an organic solvent. It is widely used for sample preparation and metabolite enrichment. The sample is mixed vigorously with the solvents (using an appropriate mass of sample and volume of solvents) facilitating the transfer of extractable endogenous metabolites from one solvent to the other [204] and following phase separation, the solvents are removed and rotary evaporated to obtain a crude extract [203].

##### Solid phase extraction

**Fig. 9:**
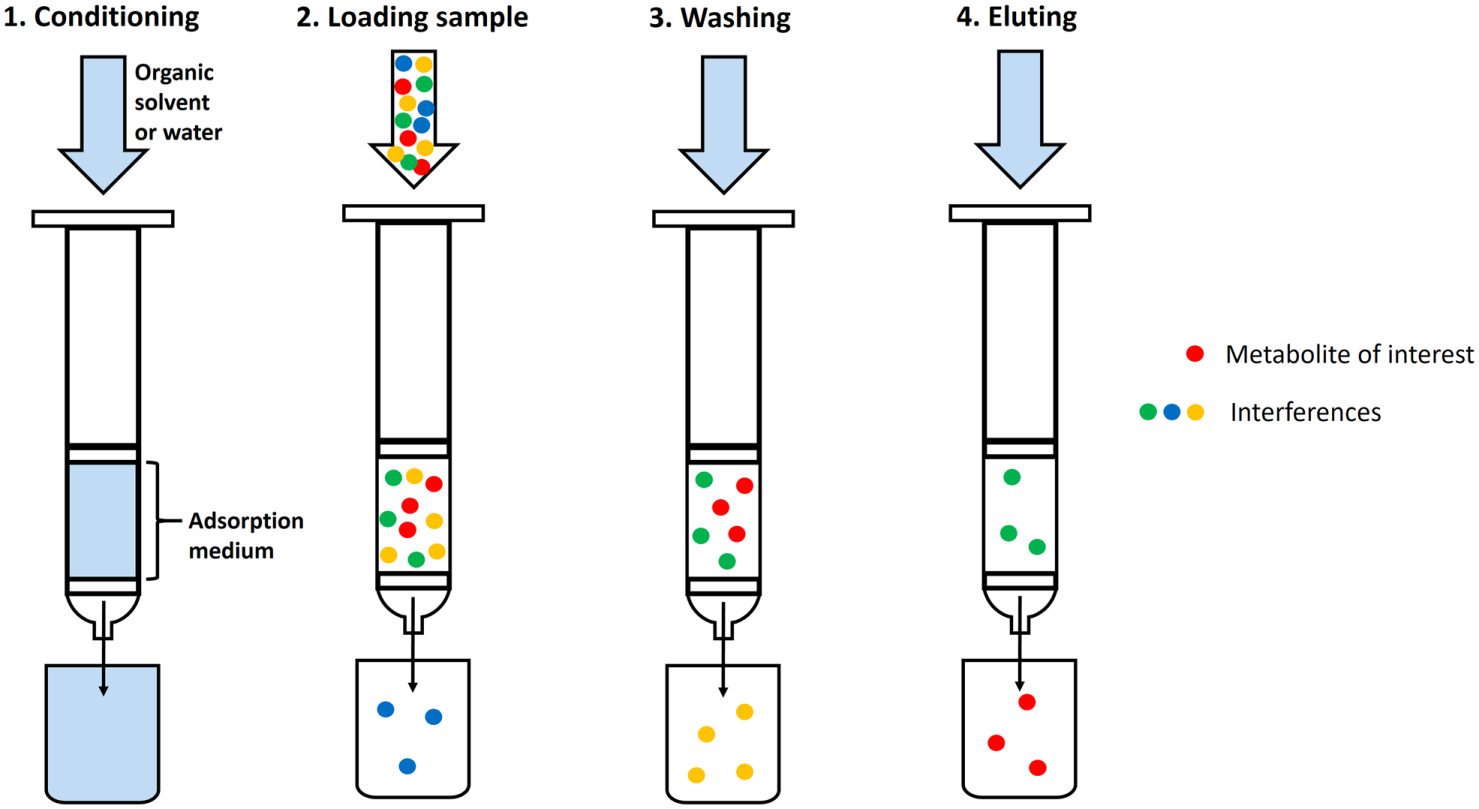
Schematic diagram of the SPE process (Adapted from [207])

Solid phase extraction is a useful technique for sample clean-up and concentration improving metabolite enrichment and analytical sensitivity (Fig. 9) [205]. It is fundamentally a miniaturised form of column chromatography; the sample is dissolved in solvent and is then passed through an open ended syringe cartridge containing adsorptive medium (solid phase) [206]. Firstly, the solid phase is conditioned by passing solvent through the cartridge—this increases the effective surface area and minimises interferences. Then the sample is loaded onto the column and the column is washed with subsequent volumes of solvent to elute undesirable/interfering compounds. The metabolites of interest are retained by the sorbent and can then be eluted with a suitable solvent [207].

Traditionally, the sorbents used include bonded silica, however newer sorbents are now available such as nanostructured material (e.g. carbon nanomaterial, electrospun nanofibers, dendrimers, and magnetic nanoparticles) and molecular recognition sorbents (e.g. aptamer-modified sorbents, immunosorbents, molecularly imprinted polymers and ion imprinting polymers [206]. SPE is cost effective, has higher reproducibility, significantly less solvent use, is easy to operate, and can be automated [205]. SPE is also amenable to preparing small volumes of samples with the ability to carry out sample preparation in 96-well plates [208].

##### Supercritical fluid extraction

Supercritical fluid extraction is a ‘green chemistry’ technique which separates compounds using a supercritical fluid instead of an organic solvent. When a liquid is above its critical temperature and pressure, it is said to be in a supercritical state and the fluid exhibits the properties of both the liquid and gas phases; diffusing into solids like a gas, but with a dissolving effect like a liquid [202]. The main supercritical fluid used is carbon dioxide (CO_2_) which is inexpensive, environmentally friendly, generally non-hazardous, and has high diffusivity and a solvent strength that can be readily optimised. Solvent-free sample recovery is also simple as CO_2_ is a gas at atmospheric conditions. SFE using CO_2_ is valuable for the extraction of metabolites which are thermally sensitive or easily oxidised as CO_2_ SFE is conducted in a non-oxidising environment and at low temperatures [209]. Thus far, the utilization of SFE has mostly been industrial and have targeted the extraction of compound classes such as alkaloids, polyphenols, polysaccharides, flavonoids, carotenoids, saponins, and fatty acids [210]. Recent applications of SFE include the isolation of carotenoids from *Capsicum chinense* (habanero peppers) [211] and *Solanum betaceum* (tamarillo) [212], vanillin and aromatic constituents from fermented vanilla beans [213], tetrahydrocannabinol from *Cannabis sativa* [214], and the recovery of lipids and pigments from algal biomass [215].

#### Analytical platforms

The main analytical platforms used for metabolite profiling are GC–MS, LC–MS and NMR, the main parameters, advantages and disadvantages of which are summarised in Table 7.

**Table 7:** Comparison of GC–MS, LC–MS and NMR. Adapted from [216]

	GC–MS	LC–MS	NMR	References
Sample preparation	Extraction and chemical derivatisation	Extraction	Generally no sample preparation necessary	[216]
Sample volume	Split: < 1 µL Splitless: 1 µL	5–20 µL	Conventional: 5–550 µL Microdroplet: ≤ 5 µL	[217–221]
Chromatographic separation	High-resolution separation	Medium-resolution separation	No separation	[216]
Sensitivity	mM–nM	mM–pM	mM–µM	[216, 222]
Limit of detection and quantification	ng–pg (10^−9^–10^−12^)	pg–fg (10^−12^–10^−15^) Low pM	Low µM	[222, 223]
Dynamic range	> 10^6^	> 10^6^	> 10^3^	[216]
Quantification accuracy	± 10%	± 10%	± 10%	[216]
Speed of analysis (per sample)	Slow (approximately 30 min)	Slow (5–9 min)	Fast (1 to 5 min)	[216]
Main advantages	High resolution High precision EI-MS library available	Soft ionisation Large mass range	No sample preparation Non-destructive Suitable for compounds which are difficult to ionise or require derivatisation	[216, 224, 225]
Main disadvantages	Significant sample preparation with chemical modification Slow analysis time Harsh ionization Limited number of molecules can be analysed	Slow analysis time	Poor sensitivity and dynamic range Some chemical classes not detected	[216]

##### GC–MS

The first component of GC–MS is the gas chromatograph which consists of a stationary phase and mobile phase. The stationary phase typically consists of a capillary column which is packed with a homogeneous solid or coated on the inside with a liquid or film [225]. The sample is volatilised in a heated inlet and the gaseous analyte molecules are carried through the stationary phase by a carrier gas such as helium or hydrogen. The separation of analyte molecules depends upon the boiling point of the compound, column temperature, and the partitioning, or distribution of each analyte molecule between the mobile and stationary phases. Firstly, compounds are separated based on their boiling points—molecules with a boiling point below oven temperature will be retained for longer—and oven temperature can be adjusted to accordingly to influence retention time. Analytes are further separated based on their individual chemistries; analytes with a higher affinity for the stationary phase are retained in the column for longer, while analytes that interact more with the mobile phase are carried through the column and elute faster [226].

The second component of GC–MS is the mass spectrometer. The analytes separated by the gas chromatograph are ionized as they enter the mass spectrometer. The most common ionisation source used in GC–MS is electron ionisation (EI) which results in the formation of high energy molecular ions with a positive charge. A large proportion of these ions undergo fragmentation depending on their structure [227]. In EI, a lot of energy is transferred to the molecules which increases the degree of fragmentation. Therefore EI is referred to as a “harsh” ionisation technique [228]. The ions are then propelled into the mass analyser (i.e. quadrupole) which detects the ion mass, abundance, and the *mass*-*to*-*charge* ratio (*m/z*) of the ions, and records this information as a mass spectrum [229]. The total ion chromatograms produced by GC–MS, and the mass spectra and fragmentation data generated can then be used to compare the chemical fingerprint of different samples (e.g. volatile natural product extracts) to putatively identify compounds using either commercially available or *in*-*house* curated natural product libraries or databases.

GC–MS is also capable of analysing small molecules (< 500 Da), and is more suited for the analysis of metabolites involved in primary metabolism such as sugars and amino acids [218, 230]. In addition, only molecules that can be volatilised without decomposition can be analysed. The high temperatures (inlet temperature 250 °C) involved in GC–MS can cause degradation of non-volatile compounds which require chemical derivatisation in order to be volatilised [231]. Therefore, compared to LC–MS, GC–MS offers limited coverage of metabolites.

##### LC–MS

LC–MS couples the separation power of liquid chromatography typically with MS based technologies to enable fast and efficient separation and putative identification of molecules which are detectable in complex mixtures such as plant extracts, and is widely used in plant metabolomics. LC–MS requires microliter volumes of extract and is suitable for the analysis of molecules with low volatility [229, 232]. The LC component of LC–MS is often a high performance liquid chromatography (HPLC) system or an ultra-high performance liquid chromatograph (UHPLC) which separates analyte molecules based on their relative affinities and interaction with a stationary phase (e.g. a C18 column) and a mobile phase (e.g. water and/or acetonitrile) [233]. The eluent from the liquid chromatograph must first be ionised. Following chromatographic separation on the HPLC, the mobile phase containing analytes is sprayed through a needle into a heated tube where the molecules are nebulised and vaporised by nitrogen. The vaporised molecules are protonated (positive mode) or deprotonated (negative mode) by a strong electric charge, forming [M+H]^+^ or [M−H]^−^ species, respectively. LC–MS typically employs a soft ionization source known as electrospray ionisation (ESI) [229, 232]. A mass analyser records the mass, abundance, *m/z* ratio, and fragmentation data for the ions. This information can then be used as mentioned above, for the putative identification of metabolites.

**Fig. 10:**
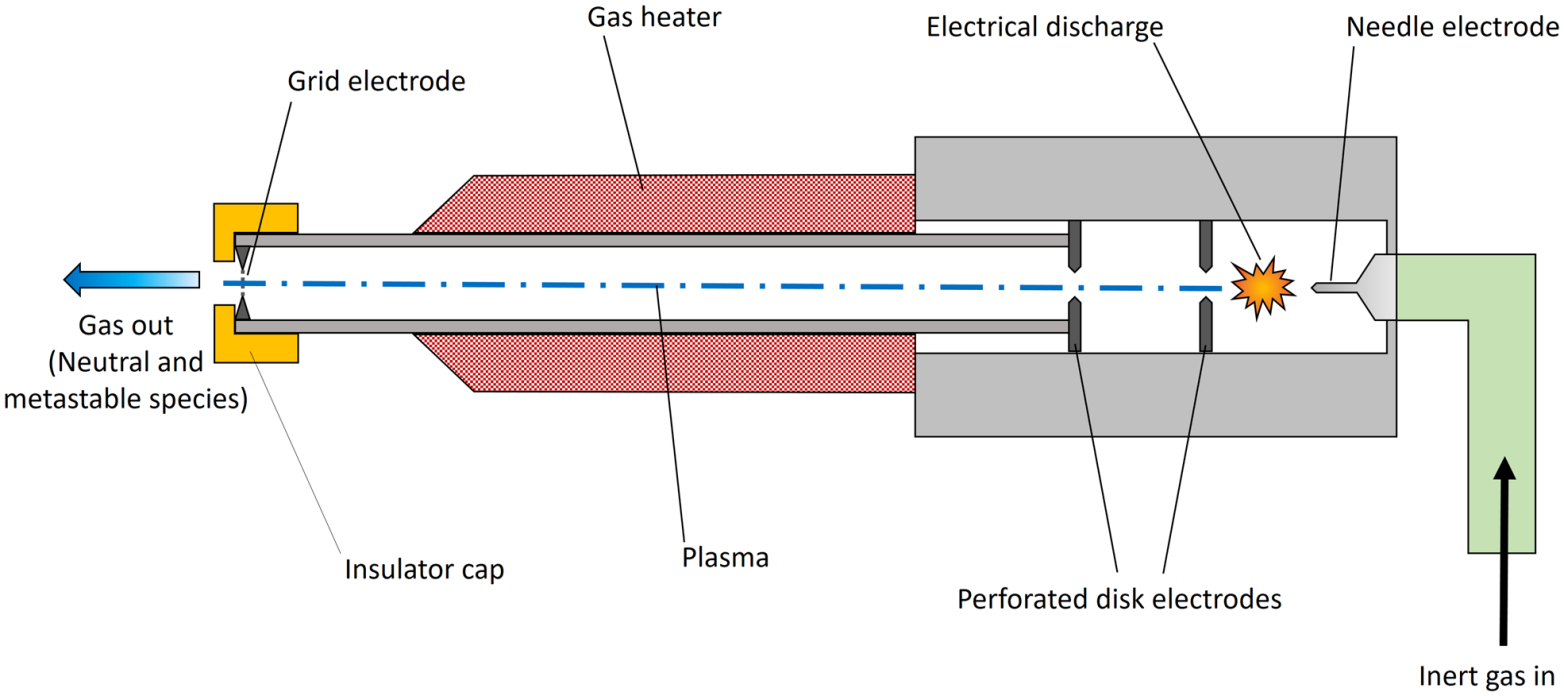
Schematic diagram of the DART™ ion source (Based on [235])

As previously mentioned, sample preparation and clean-up steps can be laborious and time-consuming processes. Direct Analysis in Real Time (DART™) is an atmospheric pressure ionisation method which permits the direct analysis of samples such as intact plant material in their native state, with virtually no sample pre-treatment required [234]. The technology was developed in 2005 by Cody and Laramee, and is based on the interaction of the sample and atmospheric gases with metastable species (i.e. long-lived electronic excited states or vibronic excited-state molecules) [235]. In the ion source (Fig. 10), an inert gas flows through a chamber in which an electrical discharge generates plasma. The plasma passes through a perforated electrode or grid electrode which remove charged species so that only neutral and metastable species exit the ion source and enter the reaction zone (located in the open atmosphere) and comes in contact with the sample surface. A grid electrode near the orifice inhibit ion-ion recombination, acts as a source of electrons by surface Penning ionisation, and facilitates ion drift towards the reaction zone. The metastable species ionises atmospheric gases and molecules off the surface of the sample upon contact which is then analysed [235, 236]. An insulator cap protects the user and the sample from high voltage [237].

DART has many advantages; rapid, real time analysis, minimal sample preparation, better ionisation efficiency, lower matrix effects, and analysis of intact sample at atmospheric pressure and ground potential. One drawback of DART is that many polar compounds are not efficiently ionised, however this can be overcome through chemical derivatisation [238]. Some applications of DART mass spectrometry in medicinal plant research include compound detection, quick quantification [239], chemical fingerprinting [240], and identification of species [241]. For instance, DART-MS has been used to quantify mitragynine, the main alkaloid responsible for the psychoactive effects of the medicinal plant *Mitragyna speciosa* (kratom) [239]. DART-MS combined with PCA has been utilised to fingerprint and differentiate between six *Rauwolfia* species based on their monoterpene indole alkaloids [240]. DART has also found extensive application in the food and pharmaceutical industries for safety and quality control. Overall, DART offers a rapid and convenient method for screening not only plants and herbal products, but also food and pharmaceuticals.

LC–MS untargeted profiling metabolomics is the preferred and an amenable analytical platform for profiling medicinal plants. This is because LC–MS is capable of detecting important classes of semi-polar compounds such as flavonoids, phenolic acids, alkaloids, and saponins, as well as those molecules which are unstable or not amenable in GC–MS [229, 232]. The broader coverage of metabolites offered by LC–MS makes it particularly appropriate for chemically profiling the rich chemical diversity of medicinal plants.

High resolution LC–MS is favoured for untargeted analysis in natural product discovery as high mass accuracy and resolution obtained with these instruments drastically improves the putative identification of metabolites by limiting the number of possible identities for each molecular feature [61]. Examples of commercial HR-LC–MS instruments include Fourier Transform-Ion Cyclotron Resonance (FT-ICR), Orbitrap™, time-of-flight (TOF–MS) mass spectrometry, and hybrids such as quadrupole time-of-flight (Q-TOF) instruments. FT-ICR determines the m/z ratio of ionised metabolites by measuring the cyclotron motion frequency of ions trapped in a stable magnetic field under vacuum [242]. The Orbitrap™ belongs to the FT-ICR family, but utilises an electrostatic ion trap rather than a magnetic field [243]. The basis of ion separation in TOF is that the total flight time of ions with the same point of origin, charge, and velocity is proportional to the mass of the ion [244]. The ions are simultaneously accelerated by a pulsed direct-current electric field through a 1–2 m long tube, which makes TOF instruments fast and sensitive as all the ions are measured at the same time [245]. Advanced TOF instruments contain an electrostatic ion mirror known a reflectron which acts to lengthen the ion flight path and thus enhance resolution [244]. Modern TOF instruments can achieve a mass resolution of up to 40,000. Orbitrap™ and FT-ICR-MS possess superior resolving power with high-field Orbitrap™ analysers achieving mass resolutions > 350,000, and up to one million in the case of FT-ICR [188].

##### NMR

Nuclear magnetic resonance is a non-destructive analytical technique which provides not only structural information of compounds, but also dynamic, and molecular interaction data [246]. NMR uses the effect of an applied magnetic field on nuclear spin properties such as the change in nuclear resonance frequency through chemical shielding, to discriminate between the different nuclei present in a molecule [246]. The structural information that can be acquired from NMR spectra include chemical shifts and spin–spin coupling constants which provide information regarding atoms, the number of ^1^H and ^13^C present and their associated chemical environment in three dimensional space. The signal intensity is an indicator of the ratio of different atoms present within a molecule. Multiplicity and integrals are useful information for determining the magnetic environment, number of hydrogens and carbons, and the connectivity of hydrogens in three dimensional space. Together, this information can be used for determining molecular structures and the proportion of different compounds in a sample [178]. One-dimensional NMR (1D NMR) is most frequently used in metabolomics studies, however two-dimensional NMR (2D NMR) methods such as ^1^H–^1^H correlated spectroscopy (COSY), ^1^H–^1^H total correlation spectroscopy (TOCSY), and heteronuclear single-quantum correlation (HSQC) makes it easier to fully characterise complex molecules by enabling better resolution of signals which may appear overlapped in 1D NMR [224].

A major advantage of NMR is the ability to perform simultaneous identification and quantification of compounds. Quantitative ^1^H NMR (qHNMR), based on the principle that signal intensity is directly proportional to the number of nuclei responsible for a given resonance frequency, is a mainstay in quality control testing and assessing purity of pharmaceuticals and herbal products [221, 247–250]. For instance, quantitative NMR has been used to quantify the antidiabetic alkaloid berberine in ‘Coptidis Rhizoma’ (an herbal drug containing the rhizomes of *Coptidis japonica*, *C. chinensis*, *C. deltoidea* and *C. teeta*) [247] and the identification and quantification of trigonelline in *Balanites aegyptiaca* fruit [248]. Trigonelline, which is also the main bioactive NP in *Trigonella foenum-graecum* (fenugreek), is recognised as a potent antihyperglycaemic compound and is now believed to be a key contributor to the antidiabetic value of *B. aegyptiaca* fruits [248]. In a more recent study, qHNMR successfully quantified the polyphenols quercitrin, ellagic acid, and gallic acid from the fruits of *Eugenia punicifolia*, a plant used in traditional medicine for treating diabetes. Quecitrin inhibits protein glycation while ellagic acid gallic acid are known to possess antioxidant properties [251]. This supports the potential health benefits of *E. punicifolia* in medicinal use [252–255].

Nuclear magnetic resonance has a number of other advantages over MS-based analytical platforms. For example, NMR does not require complicated sample preparation which is hugely time-saving. NMR is capable of discriminating between compounds with identical masses, even those with contrasting isotopomer distributions; can analyse compounds which are not easily ionised or need derivatisation for MS. NMR spectra are highly reproducible and there is a wider quantitative dynamic range [224, 256, 257]. Furthermore, as NMR is a non-destructive analytical technique, it is suitable for in vivo studies in living organisms [224]. Despite its many advantages, NMR alone cannot generally be used for the absolute molecular determination of a pure molecule. For example, proton–deuterium exchange causes some important functional groups such as carboxylic acid, phenol and amino groups unamenable to NMR detection. Therefore, NMR is often interfaced with MS to obtain complementary structural data such as molecular weight and fragmentation patterns can assist in determining the structure of new compounds in conjunction with NMR data [178–180]. For further details, please see review by Elyashberg [257].

A significant limitation of qHNMR is its low sensitivity [221]. One way of increasing sensitivity is with the use of dynamic nuclear polarisation (DNP) which utilises high frequency microwaves to transfer electron polarization to nuclear spin. The microwaves employed in DNP are in the terahertz (THz) range and are produced by electron cyclotron resonance masers (gyrotrons). THz DNP NMR has a 20–400 fold increase in sensitivity with the subsequent reduction in the amount of sample required for analysis, and reduced acquisition time making it a powerful technology for analysing complex molecules such as proteins both in solid-state and solution NMR [258].

### Metabolomics applications in diabetes research

To date, metabolomics-based approaches have become increasingly applied to all aspects of diabetes research. Recent literature shows that metabolomics has been applied to biomarker and drug discovery, drug assessment [259, 260], toxicology [261, 262], and clinical biochemistry with a particular focus on gestational diabetes mellitus (GDM) [263–270]. Some of the recent metabolomics-aided discoveries in antidiabetic plant research are summarised below.

#### Metabolomics applied to anti-diabetic plant research

Numerous evidence-based medicinal plants and their antihyperglycaemic properties have also been evaluated using metabolite profiling approaches in order to identify their active constituents, to determine their modes of action, and to evaluate their effect on metabolic pathways. For instance, Ablat [271] used LC–MS^n^ (liquid chromatography–tandem mass spectrometry) and NMR to investigate various fractions of a methanol–water extract prepared from the seeds of *Brucea javanica* (Simaroubaceae). The ethyl acetate fraction was found to lower blood glucose levels significantly and increase serum insulin in diabetic rats. Seven compounds (vanillic acid, brucein D, brucein E, parahydroxybenzoic acid, luteolin, protocatechuic acid, and gallic acid) were identified from this fraction. Out of these, luteolin was a strong inhibitor of *α*-glucosidase and glycogen phosphorylase-*α* identifying luteolin as an important antidiabetic compound. This finding shows that the alleviation of postprandial hyperglycaemia via the inhibition of *α*-glucosidase, and suppression of endogenous glucose production by glycogenolysis via the inhibition of glycogen phosphorylase-*α* are among the antidiabetic mechanisms of action of *B. javanica* [271]. Identification of bioactive fractions and/or constituents (e.g. luteolin) makes it possible to prepare extracts with improved efficacy.

**Fig. 11:**
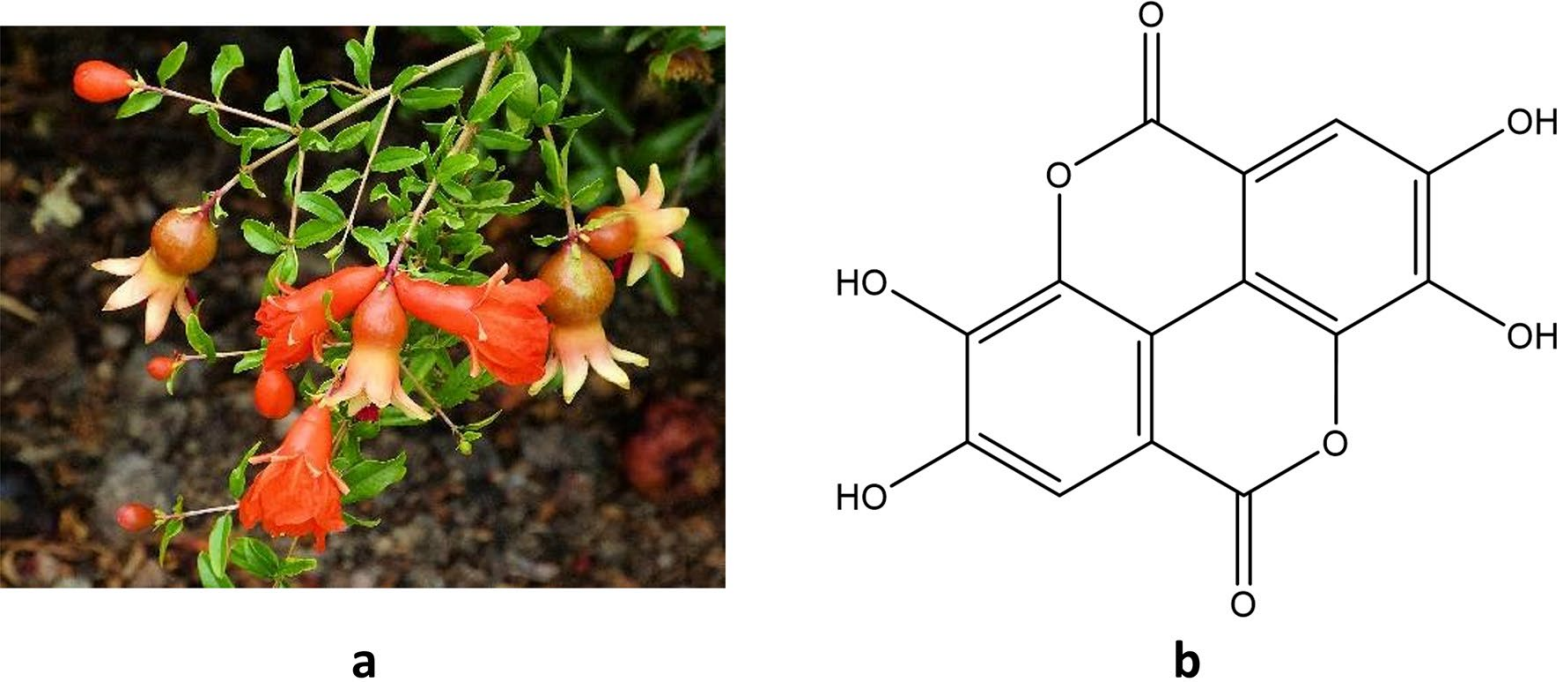
**a** *Punica granatum* plant with immature fruit,** b** Ellagic acid, the main constituent in fresh pomegranate juice

A similar study by Rouhi [272] demonstrated that ellagic acid (Fig. 11), a molecule with antioxidant properties, was the primary constituent in fresh pomegranate juice (*Punica granatum*). Pomegranate juice had no significant effects on blood glucose and plasma insulin levels of diabetic rats compared to the diabetic control group. However, pomegranate juice improved the lipid profile by causing a significant reduction in total plasma cholesterol, triglycerides, and low-density lipoprotein. Pomegranate juice also displayed significant antioxidant activity and appeared to promote pancreatic islet repair and reduce inflammation in the rats [272]. The antioxidant benefits of pomegranate juice has been attributed to ellagic acid and catechins. Fresh pomegranates or bottled pomegranate juice are readily available in most parts of the world and can easily be incorporated into the diet. Consuming pomegranate juice may assist in lowering cholesterol and benefit islet health in diabetic patients, as well as have other health benefits due to the nutritional value and antioxidant content of pomegranate juice [272].

Ma [273] reported several compounds with potential *α*-amylase inhibitory activity based on changes in the metabolite profile of *Rhodiola rosea* plant extracts pre- and post-incubation with *α*-amylase enzyme. Profiling was carried out using UHPLC-DAD–TOF–MS^n^ (ultra-high-performance liquid chromatography-diode array detector–time of flight–tandem mass spectrometry), which identified the polyphenols epigallocatechin gallate, kaempferol, and tricin. These polyphenols demonstrate free radical scavenging properties and can thus function as potent antioxidants. This is of significance as oxidative stress contributes significantly to the development and progression of diabetes and complications, therefore the intake of *R. rosea* extract may not only benefit diabetic patients control postprandial hyperglycaemia, it can also protect against the deleterious effects of oxidative stress [273].

In a study by Zilani [274] the authors used HPLC and LC–MS which lead to the detection of ellagic acid, naringenin, β-sitosterol, and p-coumaric acid in a *Pisum sativum* (peas) extract which also exhibited in vitro antioxidant properties, and improved oral glucose tolerance in diabetic mice. Sixteen compounds including caffeic acid, luteolin glucoside, xanthomicrol, carvacrol, and luteolin were putatively identified from an *Origanum glandulosum* extract via LC–MS. Although the compounds were not evaluated individually, the extract showed dose-dependent *α*-amylase inhibitory potential similar to acarbose, and antioxidant activity comparable to trolox (an antioxidant analogue of vitamin E, used as a control in antioxidant assays) [170].

#### Clinical biomarker discovery

Metabolomic studies have uncovered unforeseen changes in metabolic pathways in insulin resistant and diabetic states. Significant among these discoveries is the link between abnormalities in amino acid metabolism and insulin resistance. For example, in a study by Hernández-Alvarez [275] the authors found that branched-chain amino acids (BCAA) and branched-chain keto acids were elevated in the plasma of patients with diabetes. Notable changes in the expression of genes involved in BCAA indicated that muscle BCAA metabolism was impaired in the diabetic state [275]. Similarly, metabolite profiling of blood specimens from the Framingham Offspring study indicated a significant correlation between high baseline levels of leucine, isoleucine, valine, phenylalanine, and tyrosine, and increased risk of developing T2DM. In addition, metabolites involved in the metabolism of nucleotides, urea cycle, and methyl transfer metabolites were also highly correlated [276]. The findings relating to the urea cycle metabolites was consistent with the results of a separate study which discovered that patients with T2DM had increased plasma levels of arginine, and decreased levels of ornithine [277]. Furthermore, metabolite profiling has revealed significant differences in the levels of certain urinary metabolites such as alanine, betaine, formic acid, and trigonelline, between diabetic and non-diabetic individuals [278]. Investigating the metabolic pathways that involve these metabolites may provide new insights into the pathophysiology of diabetes and the involvement of these metabolites.

Other diabetes-related biomarkers discovered in recent years include the odd-chain fatty acid phosphatidylcholines, 2-hydroxyethanesulfonate [279], methylsuccinate, guanidoacetate, methylguanidine, and hippurate [278].

Metabolites with abnormal levels in a disease state, such as the examples above, can serve as important biomarkers for early detection of disease and/or better risk prediction. New predictive models developed using newly-discovered serum biomarkers have demonstrated higher accuracy in long-term prediction of T2DM when used alongside existing predictive models such as fasting BGLs [280]. This is important as improved predictive models enable earlier detection of individuals at risk of developing T2DM and therefore interventions can be applied sooner to mitigate the risk.

#### Diabetes complications

Metabolite profiling has also been useful towards understanding diabetic complications such as eye damage and renal disease. For example Pietrowska [281] used LC–MS to discover that several tryptophan-derived compounds (lactoyl-tryptophan, hydroxytryptophan, and hydroxyanthranilic acid) and a number of antioxidants (methyltetrahydrofolic acid, taurine, niacinamide, xanthine, and uric acid) were depleted in the aqueous humour of the eye of diabetic patients with cataracts compared to non-diabetic cataract patients. Tryptophan derivatives protect eyes from UV (ultra violet light) damage by absorbing and filtering out UV light. Therefore, it is hypothesised that the decrease in tryptophan metabolites, and increased oxidative stress due to diminished antioxidants may account for the higher risk of cataracts, glaucoma and retinopathy seen in diabetic patients [281].

Other studies have reported abnormalities in the arginase, polyol, and ascorbic acid pathways in diabetic retinopathy [282]. For example, Paris [283] detected significantly elevated levels of proline in the vitreous humour of patients with diabetic retinopathy. This is attributable to the excessive conversion of arginine to proline due to overactivity of arginase II in diabetic retinopathy, which also limits the amount of arginine available for the production of nitric oxide (NO ) via the nitric oxide synthase (NOS) pathway. The resultant reduction in NO levels leads to endothelial dysfunction and impaired vasodilation, and increased ROS generation due to NOS uncoupling causes additional damage to the retina [284]. An ^1^H NMR-based metabolomic analysis of vitreous fluid found significantly lower levels of ascorbic acid and galactitol in patients with diabetic retinopathy [285]. In diabetes, intraocular ascorbic acid levels are reduced as high glucose concentrations competitively inhibit the GLUT1-mediated transport of ascorbic acid into retinal cells. Ascorbic acid protects the eye from light-mediated free radical damage [286], and the reduced level of ascorbic acid under hyperglycaemic conditions increases the vulnerability of retinal cells to photo-oxidation [287]. Similarly, when there is an excess of glucose, the metabolism of glucose to sorbitol and fructose takes precedence over the metabolism of galactose to galactitol via the polyol pathway. The accumulation of sorbitol and fructose increases oxidative stress and has also been shown to contribute to the development of cataracts [288].

Microvascular complications of diabetes is a leading cause of diabetic kidney diseases, and a major contributor to end-stage renal failure [11]. Li [289] used a GC–MS untargeted approach to analyse patient urine samples which showed that dysregulation of mitochondrial and fatty acid metabolism in the kidneys contributed to the development of diabetic kidney disease (DKD). The study found a clear differentiation between the urine metabolite profiles of the healthy control group, T2DM group without kidney disease, and the DKD group as confirmed by orthogonal partial least squares discriminant analysis (OPLS-DA). The authors identified 33 metabolites which were significantly different between the DKD and the two control groups without DKD. These included, higher levels of uric acid, 1,5-anhydroglutcitol and hippuric acid, stearic acid, and palmitic acid and lower levels of uracil, glycine, isocitric acid, aconitic acid, 4-hydroxybutyrate, glycolic acid, and 2-deoxyerythritol in the DKD group [289]. A separate study utilising an LC–MS and GC–MS combined targeted approach identified significant dissimilarities in the levels of a number of serum metabolites in DKD patients. Several short acylcarnitines and their dicarboxylic derivatives (C2–C6) were high in DKD, pointing to abnormalities in the metabolism of fatty acids and amino acids. In addition, several phosphatidylcholines were lower while several sphingomyelin-ceramides were higher in DKD [290]. Together, these urine and serum metabolites can serve as potential sensitive biomarkers of DKD for non-invasive early diagnostics to better manage diabetes. With improved risk prediction, protective interventions can be applied early to prevent the progression of complications.

#### Metabolic effects of medications and dietary interventions

Metabolomics can evaluate the metabolic effects of medications and dietary metabolites as well as of NP extracts. For instance, a metabolomics-based study found that 90 days of treatment with metformin increased endogenous glucose production in newly diagnosed T2DM patients. This challenges the widely accepted paradigm that the antihyperglycaemic effects of metformin is primarily due to inhibition of endogenous glucose production in the liver [260]. This illustrates the importance of establishing the metabolic effects of drugs and determining whether the same drug may differ in their mechanisms of action within different stages of a disease.

An LC–MS^n^-based profiling of urine samples showed that the sulfonylurea drug glimepiride caused a reduction in the levels of cyclic adenosine monophosphate (cAMP) in urine indicating that glimepiride affects cAMP. cAMP is a secondary messenger important for insulin secretion as well as the metabolism of the amino acids: purine, tyrosine and histidine [259]. This suggested that cAMP can be a potential target for anti-diabetic therapy, and that protein metabolism abnormalities can be expected as a side-effect of glimepiride. It is not just synthetic medicines that can have side effects. GC–MS-based metabolite profiling revealed that a Chinese medicinal herbal preparation (Fu–Zhu–Jiang–Tang tablet) increased serum levels of oxalic acid [291] which is associated with an increased risk of kidney stones [292]. It is estimated that 76–86% of the population commonly use traditional medicine [293]. It is therefore important to understand any harmful effects traditional medicinal preparations may have. Overall, the above examples highlight the importance of evaluating the broader effects of antidiabetic therapies on the metabolome which in turn enhances our understanding of their mechanisms of action, side-effects and non-hypoglycaemic benefits.

Dietary modification and supplements play a significant role in diabetes therapy. Metabolomics-based animal studies have demonstrated that increased consumption of resistant starch delays the onset of diabetes. Resistant starch is a fibre-like component of dietary carbohydrate which resists hydrolysis by intestinal enzymes. Resistant starch is formed due to natural modifications to starch which occur during cooking processes such as the recrystallization of dispersed starch which take place upon cooling of cooked or boiled starchy food (e.g. rice, potatoes) [294–296]. Gonzalez-Dominguez [297] discovered that insulin-mediated metabolic disturbances were exacerbated by the co-intake of caffeine and beverages high in sugar. The authors utilized flow injection mass spectrometry and ultra-high performance LC–MS to analyse the serum metabolite profiles of healthy volunteers who either consumed regular Coke™ (caffeine + sugar), Coke Zero™ (no sugar), caffeine-free Coke™, or caffeine-free Coke Zero™ [297]. The main limitation of the study was the small sample size consisting of ten male volunteers. Others investigated the effect of red meat intake on the human serum metabolite profile and discovered significant similarities between the metabolite profile of regular consumers of red meat and that of patients with diabetes [298]. The study revealed significant differences between several classes of glycerophospholipids and lysophospholipids as well as phosphatidylcholine and sphingomyelin. Whether this implies a link between red meat consumption and diabetes is unclear.

Metabolomic studies create a wealth of information regarding changes in metabolite levels and pathways during biochemical or physiological perturbations. The ability to link biochemical parameters with physiological status enables the categorisation of diseases, risk stratification, discovery of novel predictive biomarkers and provides new knowledge on mechanisms of pathogenesis and targets for intervention or therapy. The above studies demonstrate that metabolite profiling is a valuable tool in diabetes research. In addition to metabolite profiling of antidiabetic plants, metabolomic studies create a wealth of information regarding changes in metabolite levels and metabolic pathways during biochemical or physiological perturbations. The ability to link biochemical parameters with physiological status enables the categorisation of diseases, risk stratification, discovery of novel predictive biomarkers, and provides new knowledge on mechanisms of pathogenesis and targets for intervention or therapy.

## Conclusion

Type 2 Diabetes Mellitus is a complex metabolic disorder with negative consequence on longevity and quality of life. With changing lifestyles, the prevalence of diabetes is expected to rise, and there is an increasing need for novel and alternative therapies that can help manage diabetes more efficiently, affordably, and with less side-effects. Plants have been used in traditional medicinal systems for successful treatment of diabetes and have great potential as valuable alternative antidiabetic therapies and novel drug leads. The search for antidiabetic plants and natural products rely on testing for known antihyperglycaemic mechanisms of action of current medications prescribed for diabetes. Several experimental models including animals, isolated tissue, immortalised cell lines, and biochemical assays are utilised for screening plants for antidiabetic activity. In particular, screening plant extracts for inhibition of the carbohydrases *α*-amylase and *α*-glucosidase using computer-aided molecular docking studies and biochemical assays have become popular approaches due to their amenability to high-throughput screening. Modern technologies such as HR LC–MS, MS^n^, and NMR offer powerful tools for the detailed analysis of plant extracts to identify novel bioactive molecules which can be developed into novel drugs for diabetes management.

The complex, multi-organ nature of diabetes necessitates the use of multiple experimental models as no single model can accurately portray all the pathological aspects of the disease. The availability of a range of experimental models makes it possible to select one that is appropriate for the aims of a study. Although there are several avenues for exploring antidiabetic properties of plants, there is a lack of standard protocols for most of the assays which makes it difficult to compare results between studies. Development of standardised testing methods for known therapeutic targets of diabetes are necessary and beneficial.

## Data Availability

Not applicable.

## Abbreviations

1D NMR: one-dimensional nuclear magnetic resonance
2D NMR: two-dimensional nuclear magnetic resonance
Akt: protein kinase B (also abbreviated as PKB)
ALX: alloxan
BCAA: branched-chain amino acids
cAMP: cyclic adenosine monophosphate
COSY: correlation spectroscopy
CVD: cardiovascular disease
DART™: Direct Analysis in Real Time
DIO: diet-induced obesity
DKA: diabetic ketoacidosis
DKD: diabetic kidney disease
DPP-4: dipeptidyl peptidase-4
DNP: dynamic nuclear polarisation
EI: electron ionisation
ELISA: enzyme-linked immunosorbent assay
ESI: electro-spray ionisation
FT-ICR MS: Fourier transform-ion cyclotron resonance mass spectrometry
G6Pase: glucose 6-phosphatase
GC–MS: gas chromatography–mass spectrometry
GDM: gestational diabetes mellitus
GIP: glucose-dependent insulinotropic peptide
GK rat: Goto-Kakizaki rat
GLP-1: glucagon-like peptide 1
GLUT4: glucose transporter type 4
GPR119: G protein-coupled receptor 119
GTP: guanosine triphosphate
GSH: glutathione
GSV: GLUT4 storage vesicle
HbA1c: glycosylated haemoglobin
HPLC: high performance liquid chromatography
HR LC–MS: high resolution liquid chromatography–mass spectrometry
HSD: high sugar diet
HSQC: heteronuclear single-quantum correlation
INS-R: insulin receptor
IR: insulin resistance
IRS-1: insulin receptor substrate 1
JCR/LA-cp rat: James C Russell/LA corpulent rat
K_i_: theoretical inhibition constant
KK A^Y^ mouse: KK yellow obese mouse
KK mouse: Kuo Kundo mouse
LC–MS: liquid chromatography–mass spectrometry
LLC: liquid–liquid extraction
MS: mass spectrometry
MS^n^: tandem mass spectrometry
NMR: nuclear magnetic resonance
NO: nitric oxide
NOD: non-obese diabetic
NOS: nitric oxide synthase
NP: natural product
NSY mouse: Nagoya-Shibata-Yasuda mouse
NZO mouse: New Zealand Obese mouse
OLETF rat: Otsuka Long-Evans Tokushima fat rat
OPLS-DA: orthogonal partial least squares discriminant analysis
PCA: principle component analysis
PDK1: 3-phosphoinositide-dependent protein kinase 1
PEPCK: phosphoenolpyruvate carboxykinase
PI-3K: phosphatidylinositol 3-kinase
PIP2: phosphatidylinositol-4,5-*bi*phosphate
PIP3: phosphatidylinositol-4,5-*tri*phosphate
PKB: protein kinase B (same as Akt)
PLS-DA: partial least squares-discriminant analysis
PNPG: *p*-nitrophenyl-α-d-glucopyranoside
PPT: protein precipitation
PTB: phosphotyrosine-binding
PTB-1B: protein tyrosine phosphatase-1B
qHNMR: quantitative proton nuclear magnetic resonance
Q-TOF: quadrupole time-of-flight
ROS: reactive oxygen species
RTK: receptor tyrosine kinase
SFE: supercritical fluid extraction
SGLT: sodium glucose co-transporter protein
SH2: Src-homology 2
SHR/N-cp: spontaneously hypertensive rat/NIH-corpulent rat
SPE: solid phase extraction
STZ: streptozotocin
SUR1: sulfonylurea receptor
SV40: simian vacuolating virus 40
T1DM: Type 1 Diabetes Mellitus
T2DM: Type 2 Diabetes Mellitus
TOCSY: total correlation spectroscopy
THz DNP NMR: terahertz dynamic nuclear polarisation nuclear magnetic resonance
TOF-MS: time-of-flight mass spectrometry
TSOD mouse: Tsumara Suzuki obese diabetes
UHPLC: ultra-high performance liquid chromatography
UHPLC-DAD–TOF–MS^n^: ultra-high-performance liquid chromatography-diode array detector–time of flight–tandem mass spectrometry
UV: ultra violet light
ZDF: Zucker diabetic fatty rat
ZFDM rat: Zucker Fatty Diabetes Mellitus rat
